# Overcoming challenges in implementing condition monitoring services: expert elicitation

**DOI:** 10.12688/openreseurope.20044.1

**Published:** 2025-05-29

**Authors:** Ion Iriarte, Hien Nguyen Ngoc, Ganix Lasa

**Affiliations:** 1 Design Innovation Center, Mondragon University Higher Polytechnic School, Arrasate, Basque Country, 20500, Spain

**Keywords:** Internet of Things, digital servitization, product-service systems, condition monitoring services, digital manufacturing.

## Abstract

**Background:**

The introduction of the Internet of Things (IoT) has created new opportunities for servitization in manufacturing, enabling equipment providers to deliver digital services that enhance product performance and customer value. Among these, Condition Monitoring Services (CMS) have gained prominence, particularly with Original Equipment Manufacturers (OEMs) as a means to remotely monitor machinery, predict failures, and optimize maintenance. However, despite the technical maturity of these solutions, many OEMs struggle to translate CMS into sustainable business value.

**Methods:**

This study draws on qualitative insights gathered through semi-structured interviews with 13 industry experts and academic researchers actively involved in designing, deploying, and studying CMS in manufacturing. A thematic analysis was conducted to identify recurring challenges and success factors in the commercialization and implementation of CMS.

**Results:**

Findings reveal that while IoT infrastructure is a necessary enabler, CMS success hinges on the development of complementary organizational capabilities. These include service-oriented business design, alignment of internal processes, and the co-creation of value with customers. A lack of standardized performance metrics, uncertainty around data ownership, and difficulties in building trust with clients were identified as key barriers. The study offers actionable recommendations to address these issues, including the use of transparent accountability mechanisms and adaptive service development strategies.

**Conclusions:**

This research advances the understanding of digital servitization in manufacturing by focusing specifically on the real-world implementation of CMS. It highlights the importance of integrating technological capabilities with strategic and organizational readiness. By presenting grounded insights from expert perspectives, the study provides practical guidance for OEMs aiming to transition from product-centric to service-oriented business models in the era of industrial IoT.

## Introduction

The circulation of Internet of Things (IoT) technologies such as, wireless sensor networks, big data, cloud computing, and cyber physical systems (CPSs) in Industry 4.0 ( Kalsoom *et al.*, 2020) has opened up numerous opportunities for servitization in the manufacturing sector ( Kowalkowski *et al.*, 2017). In this context, numerous researchers have explored the connection between maintenance services and values in use generated from operational activities throughout the life cycle ( Donca *et al.*, 2007; Takata, 2013). This relationship reveals a conflicting objective between production and maintenance operations. For example, on one hand, there is a strong emphasis on maximizing the production ratio of production lines, often leading to a neglect of maintenance activities. On the other hand, an increase in maintenance activities or an improper scheduling of such activities can reduce the availability of machines and result in lower productivity ratios. Consequently, maintenance entails a demanding set of tasks performed on industrial machines. As a result, the outsourcing of these maintenance tasks as services is receiving attention in the industrial business-to-business (B2B) strategy ( Gómez *et al.*, 2009; Mourtzis *et al.*, 2018; Silvestri *et al.*, 2020). This approach promotes novel service-oriented value propositions that customers might be willing to incorporate into their maintenance strategies, and Original Equipment Manufacturers (OEMs) may include in their offerings ( Hien *et al.*, 2022).

These maintenance services may encompass remote maintenance, skills training, diagnosis, upgrading, and other offerings, which can be integrated with their physical products (machines and equipment) to form Product-Service Systems (PSS). In this context, appropriate design and planning play a critical role in enabling successful maintenance services ( Holgado, 2019; Wittenberg, 2016). Particularly, OEMs must have the ability to intentionally design machinery products in a way that facilitates condition monitoring services (CMS), such as by incorporating sensors that provide reliable insights into the equipment's condition ( Slatter *et al.*, 2021) and consequently advanced maintenance services. Previous research has highlighted examples of OEMs remotely monitoring their customers' products and offering condition monitoring solutions as basis of their value propositions of advanced maintenance services ( Baines & Lightfoot, 2013; Lanzilotti *et al.*, 2022; Slatter *et al.*, 2021).

This trend leads to the emergence of servitization where OEMs are striving to distinguish themselves by offering PSS that combine products with lifecycle-oriented services ( Rymaszewska *et al.*, 2017; Slatter *et al.*, 2021). Through this approach, they aim to maximize the value in use generated throughout the entire product lifecycle for customers and then exchange for new revenue streams (e.g., revenue through use, risk and revenue sharing). Leveraging technologies such as IoT and data analytics, intelligent sensors become possible to continuously and remotely monitor the condition of products in the field ( Slatter *et al.*, 2021). This advancement facilitates the implementation of condition monitoring practices that offer better insight, but not limit to product lifecycle maintenance, and optimization services for resource usage and environmental impacts ( Kristoffersen *et al.*, 2021; Wu & Pi, 2023).

However, despite recognizing CMS's potential, many OEMs struggle to implement it successfully ( Bousdekis *et al.*, 2020; Hien *et al.*, 2022; March & Scudder, 2019). For example, previous research studies have highlighted various technical challenges in implementing CMS, such as the improper selection of critical components ( Selcuk, 2017) and the scarcity of failure data from field products ( Goyal & Pabla, 2015), among others. However, it should be noted that technical aspects represent just one of several significant dimensions in CMS implementation ( Lee, 2020a). Lee (2020b) further explains that the primary challenge in Industry 4.0, including CMS, lies not in the absence of suitable technology but in effectively generating new value propositions by synergistically combining technologies in a resource-efficient and collaborative manner. On the different view, Valtakoski (2017) and Keh *et al.* (2012) reported implementation challenges related to the company's lack of capacity and resources to deliver the planned services and inaccurate estimation of service costs.

Existing research indicates a disconnect between the current academic focus on CMS and the practical challenges faced by companies when implementing it ( Fraser *et al.*, 2015). Furthermore, recent studies stress the necessity of understanding implementation challenges that go beyond purely technological aspects, highlighting a broader perspective in the implementation of CMS ( Antikainen *et al.*, 2018; Ingemarsdotter *et al.*, 2021; Lee, 2020a; Roda *et al.*, 2018).

In this study, our objective is to examine the challenges faced by OEMs when implementing CMS as part of their value proposition to customers. To this aim, we analyse these challenges from the perspectives of 13 industry experts and researchers who possess deep knowledge, practical experience, and expertise in this field. The insights provided by these experts offer valuable perspectives on implementation aspects and practical solutions and recommendations for CMS successful implementation. Specifically, we explore the challenges perceived by experts when implementing CMS within the context of OEMs, as well as the solutions they employ to address these challenges. Additionally, we present implementation recommendations from the experts for OEMs who are planning to offer or have offered CMS unsuccessfully. Our aim is to contribute to the existing digital manufacturing servitization literature and community of practice, dedicating or study to a specific research scheme of CMS by providing grounded practical insights from the experts' perspectives.

The structure of the paper is as follows. The Theoretical background section provides an overview of CMS, reviewing the existing literature on challenges to CMS implementation. The following section outlines the research methodology employed for expert elicitation and details the framework development process based on expert inputs. In Findings section, we present the identified challenges, possible solutions, and recommendations for the successful implementation of CMS as provided by the experts, and we discuss them in relation to previous literature. Finally, in the last section, we present the paper's conclusions and suggest potential avenues for future research.

## Theoretical background

### Introduction to CMS

Maintenance practices in the context of Industry 4.0 involve the integration of IoT technologies to improve maintenance operations across life-cycle maintenance management ( Hien *et al.*, 2022; Mulders & Haarman, 2017; Takata, 1999). As a notable example GE Power leverages IoT technologies to enable live monitoring of its power plants, facilitating proactive and preventive maintenance efforts ( Paschou *et al.*, 2020). The maintenance goal is to maximize uptime by minimizing unplanned, reactive maintenance activities through assessment of physical conditions, analysis and the possible ensuing maintenance actions ( Ingemarsdotter *et al.*, 2021). A key aspect of this approach is the utilization of IoT, which goes beyond mere machine-to-machine communication by incorporating data. OEMs can harness this data by collecting and analysing it to develop and implement CMS as data-driven services. The development of CMS, according to Jardine *et al.* (2006), involves three essential phases: data acquisition, data processing, and maintenance decision-making.

In the data acquisition phase, information about the products in the field is gathered and organized. This includes collecting condition monitoring data such as vibration, acoustic, oil analysis, temperature, pressure, or moisture data, as well as event data that describes specific product situations or maintenance tasks performed. The data allow for getting valuable insights into the real-time condition of the equipment ( Bokrantz *et al.*, 2017; Werner *et al.*, 2019). Condition monitoring data can be obtained either automatically through sensors or other measurement techniques, or manually through activities like daily oil quality checks ( Ahmad & Kamaruddin, 2012). Therefore, the intentional design approach adopted by OEMs plays a pivotal role in optimizing the performance and longevity of industrial machinery.

The data processing phase involves cleaning, analysing, and interpreting the collected data. Based on the extent of data analysis involved, CMS can be classified into four distinct types: descriptive, diagnostic, predictive, and prescriptive analytics ( Baum *et al.*, 2018). Descriptive analytics focuses on understanding past events using historical data, while diagnostic analytics aims to investigate the reasons behind specific occurrences. Predictive and prescriptive analytics employ mathematical models to anticipate future outcomes and recommend optimal interventions, respectively. The term “predictive maintenance” has gained significant attention in literature, with numerous studies emphasizing its potential business opportunities (e.g., Bousdekis *et al.*, 2020; March & Scudder, 2019).

In the decision-making phase, maintenance policies are chosen, and maintenance actions are executed based on the insights derived from the data analysis, such as selecting the optimal predictive maintenance method for use context ( Tiddens *et al.*, 2023). The optimization of maintenance policies typically takes into account factors like cost, reliability, and availability ( Pedersen & Vatn, 2022). Parameters such as the degradation patterns of products, available maintenance resources, and the potential impacts of equipment downtime are considered in this process ( Alaswad & Xiang, 2017).

In this study, the term CMS is used to encompass maintenance services across these three phases that rely on data analytics at any levels. Indeed, it is key for OEMs to possess the capacity to strategically design machinery products, ensuring seamless integration with CMS in forms of PSS ( Slatter *et al.*, 2021). In addition to technological requirements (e.g., advanced sensors, data analytics capability), the strategic integration of CMS during the machinery's design phase further fosters a synergistic relationship between OEMs and customers. This process involves the utilization of digital technologies to identify the combination of products (machinery) and services (CMS) as opportune and innovative business models to monetize the IoT investments ( Paschou *et al.*, 2020; Rymaszewska *et al.*, 2017; Wang *et al.*, 2024). This paradigm shift towards service-oriented maintenance emphasizes the transformation of traditional product-centric approaches into value-driven, outcome-based services ( Mishra *et al.*, 2023). These advanced services represent new value propositions to fulfil customer requirements, which is paraphrased by Lakhani *et al.* (2014) who said “we need to move away from a traditional “box seller” mentality and toward a solution-based sales methodology focusing on the customer’s pain points”.

The outcome-driven solutions are derived from the CMS's value proposition, which enables early identification and notification of potential failures or damages, efficient scheduling of maintenance activities, streamlined spare parts planning, automated specific maintenance tasks, and effective management of after-sales maintenance services ( Castellano *et al.*, 2017; Çınar *et al.*, 2020; Jardine *et al.*, 2006; Uhlmann *et al.*, 2021). Additionally, CMS offer environmental benefits by promoting longer operational lifespans for products, optimizing product utilization, and reducing the need for transportation of maintenance personnel and spare parts ( Johansson *et al.*, 2019). Moreover, this allows for the realization of circular strategies through the implementation of lifecycle expansion and looping strategies ( Mishra *et al.*, 2023; Potting *et al.*, 2017) in general and for meeting the 3R principles: reduce, reuse, and recycle ( Kristoffersen *et al.*, 2021; Wu & Pi, 2023).

Previous studies have presented instances of OEMs effectively employing remote product monitoring and providing CMS as value-driven services ( Lightfoot *et al.*, 2011; Rymaszewska *et al.*, 2017). Specifically, a sheet metal machinery company offered operations support and optimization as part of its value proposition, aiming to reduce unit costs for customers by selling the product through a monthly fee investment (monetization through a subscription model). Similarly, the provider of power generators provided maintenance support that offer reduced risks of outage and penalty costs as a part of larger service contract (monetization through contract agreement). These monetization mechanisms through outcome-based services are well known as advanced services that is a special case of PSS ( Baines & Lightfoot, 2014). However, despite these successful examples, the adoption of CMS remains far from widespread. Many OEMs, although recognizing the potential business advantages of CMS in digital servitization, continue to encounter severe challenges when attempting its successful implementation ( Bousdekis *et al.*, 2020; Hien *et al.*, 2022; March & Scudder, 2019). The next subsection navigates in the challenges in CMS implementation identified by extent literature.

### Previous research on the challenges in CMS implementation

As already recognized by the literature, the path toward digital servitization is challenging and does not always allow manufacturing companies to realize the expected profits ( Leoni, 2019; Turetken *et al.*, 2019). This contradictory effect is called as “service paradox”. Specifically, the service paradox indicates servitized manufacturing does not succeed in developing a profitable service business to complement an existing product-oriented business ( Cheah *et al.*, 2019; Gebauer *et al.*, 2005; Kowalkowski *et al.*, 2017). This is also understood as the market success of the servitized businesses is under expected.

Previous research on the challenges leading to the service paradox has highlighted several organizational impediments that hinder the successful digital servitization in the process industry. In particular, Van de Kerkhof *et al.* (2016) argue that, despite the potential of CMS servitized by manufacturers to improve operational efficiency and reduce downtime, these CMS are hardly integrated with their customers’ existing systems (e.g., data management system, legacy machines and systems). Therefore, one of the key barriers identified is the complexity of aligning CMS technology with existing organizational processes and structures. In many cases, difficulties are acknowledged in ensuring that the right data is captured and utilized effectively, leading to what is described as "knowledge lost in data" ( Van de Kerkhof *et al.*, 2016). This issue highlights a critical gap between the technological capabilities of CMS and the ability of organizations to leverage the insights generated for decision-making and performance improvement. These findings highlight the broader challenges seen in digital servitization efforts, where the promise of IoT technology and new business models often does not translate into immediate or expected business success, thereby reinforcing the need for an exploratory and qualitative study that seeks to clarify these challenges associated with CMS implementation ( Ingemarsdotter *et al.*, 2021; van de Kerkhof *et al.*, 2016).

Moreover, as outlined before, recent authors call for a broad exploration of implementation challenges beyond purely technological aspects ( Lee, 2020a; Marcon *et al.*, 2022). Although some studies have highlighted scattered challenges in implementing CMS, most of them focus on technical challenges, such as misusing critical components ( Selcuk, 2017), lack of data in the field ( Goyal & Pabla, 2015), or improper collection of user needs when the CMS is in use ( Wu & Pi, 2023). Similarly, authors made an effort to raise solutions that are expected to enable companies to alleviate these technical challenges. For instance, the challenge related to the improper selection of critical components was addressed by Lee *et al.* (2014) who proposed proper selection methods of critical components that need to be monitored in CMS and how the captured data from these components can be processed through proposed algorithms. In order to deal with the lack of data related to user needs, Mourtzis *et al.* (2020) recommended the use of Augmented Reality (AR) technology to deliver real-time communication and data exchange between service technicians in the field and expert engineers as end users located remotely.

Nevertheless, the other stream of research calls for the attention on service development and organizational aspects as technical aspects are only a part of several key implementation requirements ( Lee, 2020a). Specifically, Valtakoski (2017) and Keh *et al.* (2012) highlighted implementation challenges related to the company's lack of capacity and resources to deliver the planned services and inaccurate estimation of service costs. Additionally, Lee (2020b) further explains that designing and delivering CMS to customers by combining technologies in a resource-efficient and collaborative manner is the primary challenge. On the recent research, Werbińska-Wojciechowska & Winiarska (2023) recommended CMS needs to be connected with new business model development where the concept of “Machine as a Service” and “Software as a Service” are useful for research in addition to technical solutions.

Therefore, despite the available research findings, the practical application of CMS continues to pose significant challenges for OEMs. The gap between the current research on the challenges, associated solutions and the realities faced by OEMs requires further investigation and exploration to bridge this divide. Above all, the present study takes a wide and exploratory perspective on implementation challenges faced by OEMs when incorporating CMS as part of their value proposition to customers. These challenges limit potential business advantages of CMS in digital servitization whose market success remains far from widespread. Some practical solutions and recommendations are therefore required to enable OEMs to overcome the challenges. Hence, the present study poses the following questions:

- RQ1: What are the implementation challenges that limit market success of CMS?
- RQ2: What are the potential solutions to overcome these challenges?
- RQ3: What recommendation should an OEM take to offer CMS successfully?

## Methods

### Research approach and question

This study being undertaken is exploratory in nature because it concentrates on a highly specialized particular domain, which is CMS implementation for OEMs in digital servitization; there has been insufficient investigation in literature up until now as stated before. As an exploratory study, it was determined that the most effective method for conducting qualitative and interrogative research was the utilization of in-depth expert interviews because the experts are those who have the specific knowledge, practical experience, and expertise in a highly tangled subject matter. This is also aligned with Meuser & Nagel (2009) and Bogner & Menz (2009) who asked to delve into tangled phenomena that can only be elucidated by individuals possessing the comprehensive practical understanding necessary to illuminate the matter being studied.

Another aspect is the scope of the study that is not intended to infer generalized results, but rather to understand and provide practical insights to academics and practitioners about how experts perceive this specialized domain (CMS implementation by OMEs in digital servitization). These expert insights offer context-rich perspectives, implementation aspects and valuable prescriptions to practitioners. Moreover, this method of expert exploitation has also been acknowledged and commonly used in PSS and digital manufacturing servitization research, including Raddats *et al.* (2022), Benedettini (2022), Gaiardelli *et al.* (2021), Naik *et al.* (2020) and Matthyssens & Vandenbempt (2008) among others. Specifically, Raddats *et al.* (2022) used expert interviews and secondary data from 20 manufacturers to study digital service innovations while Benedettini (2022) applied the same methods to explore green servitization in the single-use medical device industry. Thus, the present study employs in-depth interviews with experts as the principal means of gathering information to investigate novel and highly specific themes and offer comprehensive expert solutions overcoming challenges in CMS implementation, in accordance with the scope of the study.

### Data collection

As expressed by the prior studies regarding the use of expert interviews, important aspects of research design in these types of studies are: (i) the definition of what constitutes an expert in the topic, (ii) the sampling method, and (iii) the response bias. First, we follow the guidelines of Gläser & Laudel (2009) and Gaiardelli *et al.* (2021) to identify ‘experts’ as outlined in Table 1. Gläser & Laudel and Gaiardelli *et al.* indicate that the expert selection criteria must ensure multidisciplinary experts who specialize in relevant major fields related to the topic, in our case: Servitization, PSS, New Service Development, Service Design and Industry 4.0 (including digital manufacturing, IoT, ICT and automation). This multidisciplinary perspective is critical in our research, as CMS implementation necessitates not only a life-cycle approach but also a deep understanding of enabling technologies and business models ( Carrera-Rivera *et al.*, 2022; Iriarte *et al.*, 2023). In addition, the chosen experts were drawn from highly industrialized regions from several European countries, including Spain, Portugal, the UK, Sweden, Finland, and Germany, to ensure a geographically diverse perspective on the topic at the European level. In addition, the experts who possess considerable practical expertise spanning from 11 to 26 years, encompassing involvement in industry-specific and academic initiatives related to CMS research and market implementation. Therefore, we argue that our experts have been carefully chosen with the objective of acquiring a holistic comprehension of both the theoretical underpinnings and practical dimensions regarding CMS implementation by OMEs. This rigorous selection process ensures the incorporation of the latest and most up-to-date practical knowledge pertaining to the challenges and recommendations associated with expert-driven solutions for implementing CMS in manufacturing digital servitization real-world contexts.

**Table 1.** Expert profile.

Expert	Major fields	Business	Role	Working years
Expert #1	Software and Systems Engineering	University	Researcher	18
Expert #2	Service Design, New Service Development	University	Researcher	11
Expert #3	Servitization, PSS, Advanced Services	Business Consultancy	Senior Consultant	22
Expert #4	Servitization, Business Model Innovation	University	Researcher	19
Expert #5	Digital Manufacturing, IoT, Automation	University	Researcher	11
Expert #6	Servitization, PSS, Advanced Services	Research Centre	Researcher	23
Expert #7	Service Management, Service Operations	OEM	Service Manager	9
Expert #8	New Service Development, IoT, Automation	OEM	Head of IoT and Automation	21
Expert #9	New Service Development, IoT, Automation	OEM	Researcher in IoT and Automation	26
Expert #10	Servitization, PSS, Advanced Services	Research Centre	Head of R&D	23
Expert #11	Software R&D, Servitization, PSS	Research Centre	Head of RDI Software Development	14
Expert #12	Service Management, Service Operations	OEM	Service Manager	11
Expert #13	Service Design, New Service Development	Research Centre	Development Manager	20

Second, according to Mason (2010) and Suri (2011), the qualitative interview approach does not have strict requirements regarding sample size of experts because the expert elicitation-based studies in nature are not looking for generalized results or inferential conclusions. However, Naik *et al.* (2020) acknowledged their research limitation in terms of internal validity owing to the smaller number of 7 expert interviews they could draw upon. Therefore, here the important criteria it is not the sample size, but the saturation of interview findings from the experts that reflects a status in which no new findings is gained in the information shared from new additional interviews. To clarify this point, Guest *et al.* (2006) studied statistically a threshold in which the saturation occurred and they revealed that the first twelve interviews accounted for 92 percent of the total information shared by interviewees. This means that according to their study additional interviews after the first twelve interviews provide little in terms of further themes, insights, perspectives or information in a study. Therefore, for our study we created a list of 20 experts on CMS with purposive sampling based on the rigorous expert selection criteria and 13 of them accepted the invitation to this study ( Table 1).

Third, several actions we made are to minimize response bias and ensure credibility of the data derived from the interviews. According to Saunders *et al.* (2016), we may enhance data credibility by offering relevant information to interviewees before the interview. Therefore, before the interview commenced, we distributed an email containing information about the study, the research objective, and a confidentiality statement to each expert. The confidentiality statement indicated that the participants' identities would be kept confidential, and they were notified that the data gathered would be reported and published in research publications. We distributed the confidentiality statement through a consent form and/or recording confirmed by each expert to acknowledge their rights when sharing information in the interview.

Lastly, to facilitate our interviews, we compiled a semi-structured interview guide with open questions as a flexible interview style that closely mimics a natural conversation ( Miles *et al.*, 2013), which we found to be the appropriate method for eliciting and combining knowledge during our interviews ( Cenamor *et al.*, 2017; Raddats *et al.*, 2022). Specifically, in the first part of the interview, we presented briefly the study background, research objective, research consent and the summary of literature review along with an exemplificative company case to facilitate the interview process as recommended by Dewit *et al.* (2021). The interview guide and associated data to the interview process performed in our research is available as Extended data of this article ( Iriarte *et al.*, 2025b).

According to Benedettini (2022), interview questions should be focused on the research questions of a study. Hence, in line with what has been mentioned earlier, the primary objective of the present study was: (i) to investigate the challenges that experts perceive when implementing CMS, (ii) to uncover the possible solutions and (iii) to pose the recommendations they employ to overcome these challenges. Hence, the second segment of the interview involved questioning the participants about their opinions and viewpoints regarding three key interview questions that aimed to gather expert perspectives on comprehending the challenges linked to the recommended solutions.

These three main questions were asked first, to minimize response bias from the interviewees ( Myers & Newman, 2007), additional detailed questions for each main question were asked later to deep dive into the topic ( Schymanietz *et al.*, 2022). In the interview process, the interviewers were granted considerable flexibility to shape and expand upon the conversation. This meant that they were allowed to deviate from predetermined questions in order to delve into intriguing themes that arose during the interviews. Consequently, the interview format was adjusted and modified to some extent during the data collection phase in order to effectively capture these emerging themes ( Cenamor *et al.*, 2017; Raddats *et al.*, 2022).

In addition, we followed the recommendation of Calabrese *et al.* (2019) to interview the experts from different business organizations in separate interviews to keep the confidentiality and avoid interviewees’ reciprocal biases and influence. To increase consistency, all interviews were conducted by the authors of this paper as suggested by Benedettini (2022). The interviews were conducted in English and took place between October and November 2024 and lasted took 40–60 minutes in average. All interviews were recorded and transcribed for the next following data analysis.

### Data analysis

Based on the objective of this study, we examine and interpret unprocessed text data of experts derived from the interviews to establish novel ideas or themes in terms of challenges associated with solutions in CMS. This data analysis process is in line with Strauss & Corbin (1998)’ description: “The researcher begins with an area of study and allows the theory to emerge from the data” (p. 12), which is known as inductive analysis. The main goal of using the inductive approach is to enable research discoveries to surface from the prevalent, influential, or noteworthy themes present in unprocessed data, without being restricted by established methodologies (experimental or hypothesis testing research).

This inductive approach was successfully used in previous digital servitization studies, including but not limit to digital and information technologies (e.g., smart and connected machines) for advanced service offerings ( Cenamor *et al.*, 2017) and the constructs of servitization challenges and their relevant indicators ( Zhang & Banerji, 2017). Thus, we follow the procedure proposed by Thomas (2006) for the data analysis in inductive analysis described by Table 2.

First, we cleaned raw text data of 13 interview scripts in total in a common format (questions or interviewer comments highlighted) and then we familiarized ourselves with the raw data by reading each interview script (of 6 to 8 pages on average) multiple times, highlighting interesting phrases and noting initial ideas. Second, we separated in total of 177 relevant phrases (or ideas) that experts shared to the three main questions associated with additional detail questions. Third, we analysed these phrases further to discover links and patterns among them and then identified 30 homogeneous categories of phrases that share similar ideas. Fourth, we refined the categories further by removing their overlap and redundancy, resulting in the identification of 25 first-order themes that expressed the experts’ perspectives on the interview questions.

**Table 2.** Data inductive analysis process.

Initial reading of text data	Identify specific phrases (or ideas) related to the three research questions	Label the phrases and/or ideas to create homogeneous categories	Reduce overlap and redundancy among the categories to create first-order themes	Create second-order themes incorporating corresponding homogeneous categories
Text data include 13 interview scripts derived from 13 experts	177	30	25	8

Lastly, we mapped the first-order themes that share relevant ideas to their corresponding second-order themes, resulting relating to three main interview questions as a core to investigate novel insights about how experts perceive challenges and their potential solutions and recommendations associated with CMS implementation. The analysis process and its results that leads to the first and second order themes is represented in Findings section.

In summary, we believe that the applied research methodology is appropriate for the purpose of the study, which addresses a highly specific and under-researched topic: CMS implementation by OEMs in digital servitization. By employing in-depth expert interviews with carefully selected experts, the study leverages the insights of research and industry professionals with extensive, multidisciplinary experience across servitization, PSS, and Industry 4.0 for CMS saucerful implementation. The rigorous expert selection process, based on well-established criteria, coupled with purposive sampling and insights saturation-based justification, strengthens our study’s credibility. Moreover, careful design of semi-structured interviews, performed and transcribed by the authors steps to minimize response bias, and the systematic use of inductive thematic analysis further enhance our methodological robustness, enabling the emergence of grounded insights directly from expert data as valid results of our exploratory purposes.

## Findings: challenges, potential solutions, and recommendations for CMS implementing

In this section we present and discuss the connections and the implications of our findings. To this aim, we structure the section according to our three main research questions and we follow the themes categorization presented in the Research methodology section ( Table 2) for each main question. Thus, the insights are divided in Table 3 for challenges, Table 4, Table 5 and Table 6 for solutions and Table 7 for recommendations.

### Challenges in CMS implementation

Table 3 illustrates the performed analysis of interview data, with the categorization of the topics in second and first-order themes as well as illustrative quotes retrieved from the experts related to the implementation challenges that limit market success of CMS. The analysis identified three second-order themes related to the implementation challenges that hinder the market success of CMS: (i) CMS value propositions, (ii) CMS design processes, and (iii) CMS requirements.

**Table 3.** Inductive analysis of interview data for CMS implementation challenges.

Illustrative examples of expert quotes	First-order themes	Second-order themes	RQ1: challenges
‘As the margin (of the CMS value proposition) is small the customer would not accept the change […] customers are often risk-averse, especially when the perceived benefits of adopting a new CMS solution do not significantly outweigh the costs or disruptions associated with the transition’ (Expert 8) ‘Some customers see the value in the CMS offering but are reluctant to buy it, often because the CMS solution fails to integrate into their operations or requires significant changes to their current setup. Even when the benefits are apparent, the complexity of reconfiguring systems and retraining personnel can deter adoption.’ (Expert 1)	Lack of increment in the value proposition	CMS value proposition	Market success of CMS value propositions is under expected
‘Every customer is unique, and it is not uncommon for us to misinterpret or incorrectly identify their requirements.’ (Expert 7) ‘If they (the OEMs) don’t think on the final users at the customers’ facilities, recognizing the value provided by the CMS would be problematic.’ (Expert 2)	Lack of holistic value proposition for different stakeholders’ perspective		
‘One challenge we face is the inability to present clear numbers or KPI translations. How can the value proposition of the CMS offerings be effectively measured?’ (Expert 7) ‘Our CMS value propositions should be expressed in measurable terms such as figures, financial KPIs, or monetary values […] currently, we lack a clear understanding of the cost implications for our customers, such as the financial impact of machine downtime per hour. While we have valuable technical insights, we are missing expertise in pricing strategies and building robust business cases.’ (Expert 8)	Lack of quantification in the CMS value proposition		
‘CMS can emerge either due to customer demand or as a proactive initiative from the company—essentially, a 'demand pull' or 'supply push' approach. Often, customers expect certain CMS to be included as part of the offering from the machinery manufacturer for free.’ (Expert 6) ‘We need to better communicate with customers to validate our assumptions. If we skip market analysis, we might design services that customers do not need, or are not ready to adopt.’ (Expert 4) ‘I think improper segmentation happens when we assume all customers operate the same way. The company needs to better communicate with different customer types to check these assumptions. Not all customers are ready or capable of implementing certain solutions in their operations.’ (Expert 9) ‘Companies appears to assume that the same service will fit all customers, without considering their unique needs. It seems unclear how the service development process involved customers.’ (Expert 2)	Improper market analysis and customer segmentation	CMS design process	
‘Some customers are not ready for things such as preventive or predictive maintenance. Moreover, to enable preventive maintenance, they need to share data with us by uploading it to our cloud. Many customers are unwilling to share their data and are hesitant to buy these offerings.’ (Expert 8) ‘Access to data is a high problem since many customers hide data because of privacy issues and lack of trust on their providers.’ (Expert 1)	Inadequate assessment in relation to access to client’s resources		
‘There is a mismatch between the service provider’s solutions and the customer’s capacity to adopt them, particularly in terms of infrastructure, IoT systems, and human resources. The solution offered cannot always be implemented within the customer’s current setup.’ (Expert 13) ‘Some solutions require significant integration across various parts of the product life-cycle, but companies face challenges in aligning their systems, infrastructure, and processes to make this possible.’ (Expert 9)	Lack of readiness in Integrability	CMS requirements	
‘If something works, they stick to it and say, ‘ok, yes your idea is attractive but let’s keep it like this; it’s working.’ They are hesitant to implement new changes, even if the new solution has potential advantages.’ (Expert 7) ‘The company did not train or hire new people and instead relied on their conventional staff to start the deployment of CMS. This approach creates limitations because the existing employees often lack the necessary skills or expertise to deliver advanced services effectively. Without proper training or recruitment of specialized personnel, the quality and scalability of the services are compromised, leading to inefficiencies and a failure to meet customer expectations. (Expert 9).	Lack of readiness in organizational changes		
‘If a critical component breaks, the customer may have to wait up to six weeks for a replacement. We know that such components fail every two to three years, therefore we need to convince customers of the importance of investing in predictive maintenance. Without it, the impact of downtime could be significant, however some customers fail on understand that.’ (Expert 8) ‘I think that the point is not about condition monitoring technology, but about how big will be the change that customers need to make to their existing routines. If the required change is too significant, they are often unwilling to implement the solution, even if it offers advantages.’ (Expert 1)	Lack of readiness in customer change management		

Notably, aligned with recent literature in the topic ( Lee, 2020a; Lee, 2020b; Murtaza *et al.*, 2024) the challenges highlighted by the experts go far beyond the technical aspects of condition monitoring technology systems. This suggests that, according to the interviewees, European OMEs’ CM technologies have already reached a sufficient level of maturity to serve as reliable and safe platforms for advanced maintenance services delivery, which aligns with the paradigm shift recently indicated by Murtaza *et al.* (2024). Instead, the challenges underlined by the experts primarily relate to integrating CM technologies in a resource-efficient and collaborative manner ( Lee, 2020b) within the CMS value proposition and customers’ needs. This view is also linked to recent studies in digital servitization that emphasize that for successful use of digital platforms in enabling advanced services requires more focus on integration and ecosystem collaboration with customers ( Jovanovic *et al.*, 2022; Kohtamäki *et al.*, 2019).

Regarding CMS value propositions, the lack of clear incremental value in the new service proposition compared to the existing status quo remains a critical issue. This is consistent with the "service paradox" noted by Gebauer *et al.* (2005) and more recently by Kamp *et al.* (2023) where investments in digital servitization often fail to translate into significant financial returns due to insufficient value demonstration for the customer. In this sense, a key pain point according to the experts lies in the absence of quantifiable metrics (in particular financial metrics), such as downtime reduction or cost precise savings, reinforce findings in recent servitization literature that underscore the importance of clear performance indicators ( Kamp *et al.*, 2023). Similarly, experts also indicated challenges of heterogeneity and data quality in maintenance decision-making. This has been detailed in studies focusing on predictive maintenance strategies, which emphasize the role of robust data analytics frameworks in tackling complexity and variability in real-world systems ( Jardine *et al.*, 2006) and it is also aligned with the transition to outcome-based models, as explored by Visnjic *et al.* (2017) that highlights the necessity of creating value through accountability and synergy across diverse service components.

In terms of CMS design processes, the challenges identified by the experts align with recent observations in digital servitization literature that stress the need for agile and iterative, customer-focused design approaches ( Iriarte *et al.*, 2023; Nguyen *et al.*, 2022a; Sjödin *et al.*, 2020). In particular, experts notably underlined challenges early in the service design process, where inadequate market analysis and customer segmentation often result in a misalignment of CMS with specific customer needs. This has been also identified as a common pitfall not only in PSS design literature but also in more general digital servitization literature ( Kohtamäki *et al.*, 2019; Naik *et al.*, 2020).

Finally, on the CMS requirements side, the lack of readiness in terms of integrability poses significant challenges, particularly in aligning CMS solutions with existing customer infrastructure and human resource capabilities. This is in part reflected in studies that underscore the need for modular, scalable solutions to enable seamless integration and adaptability ( Cenamor *et al.*, 2017; Jovanovic *et al.*, 2022). Moreover, resistance to organizational change, particularly among untrained or unmotivated staff, aligns with studies developed by authors such as Kohtamäki *et al.* (2019) and Kamp *et al.* (2023), where technology adoption for digital servitization is hindered by cultural and organizational inertia both in provider and customer organizations. Lastly, experts indicate lack of readiness in customer change management, including resistance to altering routines or understanding the benefits of CMS offerings. This aligns with Jovanovic *et al.* (2022) who noticed that insufficient collaboration and a lack of structured processes for handling change can hinder the effective implementation of digital servitization. In summary, experts stressed for unsuccessful CMS implementation the lack of clear financial performance metrics, customer and user focused design for modular, scalable solutions, highlighting challenges like data quality, internal and external resistance to change, and inadequate market alignment.

### Potential solutions on the challenges

Table 4, Table 5 and Table 6 illustrate the performed analysis of interview data, with the categorization of the topics in second and first-order themes as well as illustrative quotes retrieved from the experts related to potential solutions to the implementation challenges ( Table 3) that limit market success of CMS by OMEs. Following the finding outlined in Table 3, Table 4 refers to the solutions given by the experts regarding (i) CMS value proposition challenges, Table 5 refers to solutions related to (ii) CMS design process challenges, and Table 6 refers to solutions regarding (iii) CMS requirements challenges.

**Table 4.** Inductive analysis of interview data for solutions on CMS value proposition challenges.

Illustrative examples of expert quotes	First-order themes	Second-order themes	RQ2: solutions
**Robust value proposition based on clear benefits, easy integration in current customer systems and shared data that justify the investments.** ‘To effectively convince customers, the value proposition must be structured in a clear way. It should first highlight the tangible benefits that the solution offers, ensuring these are immediately evident. Next, the solution must demonstrate easy integration into the customer's existing systems, minimizing disruption and complexity. Additionally, concerns around data sharing should be addressed, emphasizing transparency, security, and mutual value. Finally, the proposal should justify the required investment by showcasing a strong return on investment.’ (Expert 1). ‘For any solution, the perceived benefits must outweigh the associated costs or investment. Customers need clear evidence that the solution will deliver measurable value, ensuring a strong return on investment that justifies the financial and operational commitment.’ (Expert 13).	Robust value proposition	CMS value proposition	Solutions to CMS implementation challenges
**Design for CMS value propositions in modular packages** ‘Instead of overwhelming the customer with a complete, all-encompassing solution upfront, it is more effective to offer an initial, easy-to-implement level of service. This iterative, step-by-step approach allows customers to gradually experience and recognize the value of the service while minimizing risk and uncertainty. By building trust and demonstrating incremental benefits, OEM can progressively introduce more advanced offerings, ensuring the solution aligns with the customer's evolving needs and capabilities.’ (Expert 13). ‘We have recently adopted an alternative approach by encouraging customers to start small, rather pushing them to start purchasing the entire package. We can start at the beginning with a limited or more affordable set of functionalities. This modular approach not only reduces the initial investment and perceived risk but also allows customers to experience tangible benefits early on. As their confidence grows and the value of the solution becomes clear, they might gradually scale up to more comprehensive functionalities […] one of the customers we are engaging with has already embraced this strategy.’ (Expert 12).			
**Go deeper on what customer needs in a systematic way** ‘You need to thoroughly understand the client's perspective in a systematic and structured manner, particularly in relation to their production methods. This ensures that decisions are based on accurate, objective insights rather than relying on subjective assumption.’ (Expert 2). ‘The approach should actively involve customers at every stage of the CMS development process, enabling their requirements to be effectively identified and understood.’ (Expert 4).	Holistic value proposition for different stakeholders’ perspective		
**Design for service offerings that support customer value creation also in a strategic level** ‘CMS should not only address immediate operational needs but also contribute to the customer's long-term vision, they must deliver measurable economic and operational value.’ (Expert 3).			
**Think about and work with different stakeholders of the customers** ‘The involvement of stakeholders typically includes customers’ managers or coordinators, but additionally, customers’ operators and technicians, who are the end-users directly interacting with the service, should be included to provide practical insights and technical requirements necessary to implement and integrate the service effectively within the customer’s existing infrastructure.’ (Expert 1). ‘It’s critical to engage a variety of stakeholders across the customer organization, including decision-makers, users, and technical teams, to ensure the service design fits their needs.’ (Expert 2).			
**Closer relationship with customers’ needs to be built: from transaction perspectives to partnership perspectives** ‘It’s essential to establish deeper ties with customers, moving from a focus on sales transactions to a broader, value-based partnership approach.’ (Expert 3). "True partnerships are built when service providers embed themselves into the customer’s strategic direction, working collaboratively toward shared success.’ (Expert 4).			
**Include a clear and straightforward business case, such as an easy-to-understand financial analysis or ROI calculation** ‘OEMs must build financial models into their CMS designs, providing straightforward ROI calculations to make the value clear to the customer.’ (Expert 3). ‘Clear financial analysis, highlighting ROI, helps to overcome resistance and builds confidence in the proposed solutions.’ (Expert 4).	Quantification of CMS offering		
**CMS propositions must be quantified using figures, metrics, KPIs and financial term** ‘CMS value propositions must be rooted in numbers: lifecycle cost reductions, productivity improvements and downtime mitigation metrics.’ (Expert 13) ‘Focus on data-driven metrics like energy savings, maintenance costs avoided, and operational efficiency improvements to build trust.’ (Expert 12)			

Regarding CMS value proposition, 9 first-order themes were identified by the experts. The first theme in overcoming CMS implementation challenges lies in crafting a robust value proposition. Experts emphasize that such a proposition must articulate clear benefits, including easy integration into existing customer systems and addressing concerns around data sharing. This aligns with findings by Baines *et al.* (2017), who highlight the role of a clear value proposition in mitigating customer uncertainty and enhancing trust during servitization transitions. In particular, the value proposition must provide a compelling justification for investment, showcasing measurable returns. Customers need quantitative evidence that the solution will deliver tangible benefits that outweigh its costs. Expert underline that by demonstrating transparency, security, and mutual value, the solution can build trust and encourage adoption. This aligns with the work of Kamp *et al.* (2023) who emphasizes the importance of addressing perceived risks in data sharing and demonstrating quantitively the financial viability of advanced service solutions.

Another solution outlined by the experts involves designing CMS offerings in modular packages. A modular approach introduces smaller, easily implementable services first. This step-by-step method allows customers to gradually experience the value of CMS without significant initial risks ( Dewit *et al.*, 2021). Over time, as trust is established and customers become more familiar with the offerings, OMEs can progress to more advanced and comprehensive functionalities. The concept of modularity is supported in digital servitization literature too. For instance, Bokrantz *et al.* (2017) argue that breaking solutions into modular offerings enables easier adoption by reducing complexity and lowering the initial commitment. Similarly, Matthyssens and Vandenbempt (2008) highlight that incremental service additions help firms navigate customer resistance. This iterative strategy aligns with evolving customer needs and capabilities, creating a scalable pathway to full implementation.

Understanding customer stakeholders’ needs in a systematic manner is also highlighted by the experts. Experts encourage co-design approaches by involving customers actively throughout the CMS development process. This involvement ensures that the solutions are tailored to real-world requirements rather than assumptions. By considering the perspectives of diverse stakeholders—ranging from decision-makers to operators and technicians—CMS providers can develop holistic value propositions that address both operational and strategic objectives. Recent advancements in digital servitization emphasize the importance of stakeholder engagement. For example, Iriarte *et al.* (2023) stress the value of aligning services with the needs of diverse actors within a client organization to ensure solutions are operationally feasible and strategically aligned. However, the topic of co-design approaches with customers in servitization remains significantly undertheorized in the literature.

In addition, experts stress too the importance of incorporating metrics such as lifecycle cost reductions, productivity improvements, and operational efficiency gains into the value proposition. Clear financial analysis, including ROI calculations, enhances transparency and builds confidence among customers. By using data-driven metrics, CMS providers can substantiate their claims and demonstrate the economic and operational advantages of CMS solutions. For example, Jardine *et al.* (2006) discuss the role of condition-based maintenance in quantifying tangible benefits such as reduced downtime, a critical factor for gaining customer trust and commitment.

In terms of the CMS design process, an iterative and adaptive design approach ( Sjödin *et al.*, 2020) is recommended by the experts. Interviewees highlight the need to continuously refine services based on customer feedback and evolving market needs. The iterative approach is supported by insights from Ahmad and Kamaruddin (2012), who highlight the significance of continuous refinement in maintenance strategies to enhance operational relevance and efficacy. In opinion of the experts, implementing this approach can ensure that CMS offerings remain relevant and effectively address emerging changes.

**Table 5.** Inductive analysis of interview data for solutions on CMS design process challenges.

Illustrative examples of expert quotes	First-order themes	Second-order themes	RQ2: solutions
**Iterative and step-up design approach** ‘Iterative design ensures that services are refined continuously based on feedback and evolving customer needs.’ (Expert 2) ‘Iterative development is essential to adapt services to real-world requirements as they emerge.’ (Expert 5)	CMS design process	CMS design process	Solutions to CMS implementation challenges
**Understand your business case in terms of supply push or demand pull** ‘A thorough market study is essential to distinguish between addressing current market needs and introducing innovation that customers may not yet recognize.’ (Expert 10) ‘Identify whether your service addresses a market gap (demand pull) or introduces a new concept that requires cultivating awareness (supply push).’ (Expert 13)	Proper market analysis and customer segmentation		
**Establish connections with market associations, as they have valuable insights into customers and the** **market dynamics** ‘Collaborating with market associations can provide critical insights into customer behaviour and overall market trends.’ (Expert 1) ‘Establishing strong links with these organizations can provide insights that would otherwise require significant time and resources to uncover.’ (Expert 6)			
**Conduct a market study to gather insights and understand market needs. Use this information to build** **trust and credibility with customers** ‘Effective market analysis involves gathering insights directly from customers to ensure services are designed to address real problems.’ (Expert 6) ‘When customers see their concerns reflected in the design of services, they are more likely to trust the provider.’ (Expert 11)			
**Segment clients strategically, focusing on those where you can pioneer and demonstrate the value** **of the proposition. This approach allows to create successful case studies and proof points that can** **effectively convince and attract other potential customers** ‘Demonstrating success with a focused group of clients builds credibility and encourages others to follow.’ (Expert 1) ‘Select segments where your value proposition can deliver immediate and measurable benefits, creating strong testimonials.’ (Expert 12)			
**Begin by targeting customers who are innovative, technologically advanced, and interested in adopting** **new technologies as early adopters** ‘These early adopters are often the first to see the value in your offerings and help build credibility in the market.’ (Expert 2) ‘Innovative customers serve as ideal partners for piloting and showcasing new solutions. (Expert 3)			
**Benchmark which technology convince more customers to allow access to their sources in general** **(equipment's, facilities people, processes, etc.)** ‘OEMs need to show how their solutions align with security standards and industry best practices to ensure customers feel confident in granting access to equipment and processes’. (Expert 3) ‘Showcasing how security measures meet industry standards can build trust, encouraging customers to share their facilities, processes, and data’. (Expert 5)	Proper assessment in relation to access to client’s resources		

In addition, a thorough market analysis ( Nguyen *et al.*, 2022a) in the CMS design process is also crucial according to the experts. OEMs must understand whether their services fulfil existing demand (demand pull) or require cultivating new market awareness (supply push). Moreover, collaborating with market associations and conducting pilot programs with early adopters, further aids in building credibility and trust to other potential customers, showcasing the practical value of CMS. In this sense, in line with Kohtamäki *et al.* (2019), experts emphasize that pilot implementations with innovative customers not only validate service effectiveness but also provide critical insights for iterative improvement and adoption of unfamiliar technologies.

**Table 6.** Inductive analysis of interview data for solutions on CMS requirements challenges.

Illustrative examples of expert quotes	First-order themes	Second-order themes	RQ2: solutions
**Propose modular service packages that begin with simpler offerings to minimize significant changes for the** **customer. Gradually introduce more complex, sophisticated, and higher-commitment levels** ‘The service designer may propose small services to avoid the big changes inside the customer until the whole advanced service can be implemented […] the service could be modular’. (Expert 4) ‘Use a step-up approach to gradually progressing through iterative stages of development. Start with smaller, simpler implementations and refine them over time based on feedback and results. By doing so, OEMs can avoid committing excessive resources upfront, ensuring that investments are aligned with proven outcomes and minimizing risks’. (Expert 6).	Readiness in Integrability	CMS requirements	Solutions to CMS implementation challenges
**Conduct internal pilot programs and enable the service unit to operate more independently from other business** **units within the company** ‘We should start with small-scale testing or pilot projects before full-scale implementation to ensure adaptability and minimize customer resistance to large-scale changes’. (Expert 12) ‘Establishing a dedicated team responsible for the development and delivery of services is essential. This involves allocating resources specifically for their activities. They should focus on refining and testing services within the company before external deployment, ensuring that this service team operates with a sufficient autonomy to adapt effectively’. (Expert 13)			
**Financial skills need to enhanced and financial model needs to be built** ‘As a service manager I need to develop some financial skills. For example, accounting skills are needed to help measure the costs incurred and how to determine the financial implications of having a machine down for one hour’. (Expert 12) ‘Showing the savings, benefits, and cost analysis. We need some house training in the financial department to understand and how to convert the services into the money’. (Expert 8)	Readiness in organizational change		
**Train and hire new people with service profiles** ‘When hiring new people, the focus should not solely be on individuals with IT profiles. Instead, it is essential to prioritize candidates who possess strong service-oriented skills Greater investment is needed in developing skills that complement and enhance the delivery of services’. (Expert 6). ‘We have established a new department dedicated to the design and development of services. This department operates in close collaboration with the production unit to ensure integration our service offerings with existing operational processes. A key strategy within this initiative has been to create a multidisciplinary team by combining digital experts, with production staff’. (Expert 8).			
**Carry out pilot implementation with successful business cases for customers** ‘We try to first define and develop the project completely with one client and then launch it on the market. Most of the customers do not know how to use the data’. (Expert 12) ‘We conducted pilot projects with some customers to address the challenges we had effectively. These pilots were initiated with customers who were early adopters. Their willingness to embrace innovation allows us to showcase the capabilities and benefits of our condition monitoring systems. The success of these pilot implementations serves as tangible evidence of the value our solutions bring, helping to build trust and confidence among other potential customers’. (Expert 7).	Readiness in customer change management		
**Offer financial support to customers for implementation** ‘Some customers do not have money to acquire CMS. In such cases, offering financial support—such as flexible payment plans or other incentives—could encourage adoption and help bridge the gap between the perceived value of the service and the customer's willingness to invest’. (Expert 10). ‘Customers hesitate to adopt services because of the high initial investment. Introducing financial options, such as pay-per- use or subscription models, can reduce this barrier and make services more appealing’. (Expert 6)			

Readiness for organizational change appears as a critical theme for CMS requirements. As it has been mentioned previously, this involves proposing modular service packages that begin with simpler offerings to minimize significant changes for the customer and gradually introducing more complex, sophisticated, and higher-commitment service levels. To do so, CMS providers should conduct internal pilot programs and enable the service unit to operate more independently from other business units within the company ( Kamp *et al.*, 2023; Murtaza *et al.*, 2024). Related to that, and linked to readiness in organizational change, enhancing financial skills within service teams, creating financial models to quantify costs and benefits, and training or hiring individuals with service-oriented profiles is also recommend by the experts.

Experts propose to establishing multidisciplinary teams that combine IoT expertise with operational knowledge ensures seamless integration of CMS into existing workflows. The necessity of multidisciplinary teams is mirrored in literature for example in the work of Paschou *et al.* (2020). Finally, and regarding to readiness in customer change management, according to the experts, starting pilot implementations with successful business cases provide tangible evidence of CMS benefits, building confidence among broader customer bases. Additionally, offering financial support, such as flexible payment plans or subscription models, helps lower barriers to adoption and increases the interest on CMS offerings.

### Expert recommendations

Finally, Table 7 illustrates the performed analysis of interview data, with the categorization of the topics in second and first-order themes as well as illustrative quotes retrieved from the experts related to recommendations to overcome the implementation challenges that limit market success of CMS. The experts raise the recommendations to overcome CMS implementation challenges into 2 second order-themes; recommendations on the design of the CMS and change management recommendations.

**Table 7.** Inductive analysis of interview data for recommendations to overcome CMS implementation challenges.

Illustrative examples of expert quotes	First-order themes	Second-order themes	RQ3: recommendations
**To support the business case and provide a detailed ROI analysis in a spreadsheet** ‘To design a proposal that highlight benefits, ensure easy integration, and address shared data concerns. Most importantly, it should provide clear a quantitative evidence of investment returns, supported by a structured business case.’ (Expert 1) ‘To emphasize quantitative data in order to demonstrate the value of the service, particularly how they enhance machine availability […] provide clear evidence of economic benefits and a strong ROI to ensure that customers are confident and convinced’. (Expert 3)	Robust value proposition	Design for CMS	Recommendation to CMS implementation challenges
**To establish a systematic iterative design process to design for both customers’ strategies and operations** ‘To develop and iterative service design for adapting services to real-world requirements as they evolve so CMS offerings align with both the strategic goals and operational needs of the customer, providing continuous refinement and relevance throughout the implementation process’. (Expert 4) ‘To use a step-up approach design process to gradually progress through iterative stages of new service development is key’. (Expert 6)	Systematic CMS design methodology		
**To capture customers’ needs systematically through close relationship and cooperation** ‘To stablish an approach to actively involve customers at every stage of the CMS development process, enabling their requirements to be effectively identified and understood as soon as possible.’ (Expert 4) ‘To get involved in the customer field operations to understand their strategy.’ (Expert 7)	Deep understating of customer reequipments		
**To get the right people inside customers’ entire life-cycle** ‘To determine which stakeholders are involved at each stage. This includes managers to define benefits, but also IT personnel, operators and technicians who directly use the service, and familiar with the processes.’ (Expert 1) ‘To get the right people inside customers entire life-cycle service development, also in the phase of prototyping.’ (Expert 12)	Holistic network of Stakeholders		
**To carry out research to understand suitable business models** ‘To identify whether your service addresses a market gap or introduces a new concept that requires cultivating awareness to guide the business model design to align with market dynamics effectively.’ (Expert 13) ‘To collaborate with market associations to identify critical insights into customer behaviour and market trends for shaping a business model that aligns with the target audience.’ (Expert 3)	Proper monetization models		
**To test and make prototype for small pilot implementations in customers** ‘To create prototypes and run small-scale tests to fine-tune solutions and ensure alignment with customer needs before large-scale rollout.’ (Expert 4) ‘To start with small-scale testing or pilot projects before full-scale implementation to ensure adaptability and minimize customer resistance to large-scale changes.’ (Expert 13)	Prototyping of CMS		
**To create an independent department to design and run CMS** ‘To establish a new department dedicated to the design and development of services who operates in close collaboration with the production unit.’ (Expert 8) ‘To establish a dedicated and independent team responsible for the development and delivery of services to ensure adaptability so that customer strategies and operations are effectively supported.’ (Expert 13)	Internal change	Change management	
**To take into account the changes required from customers** ‘To understand the internal routines of the customer and how much change they will need to make to adopt the service. If the service requires them to change too much, they might resist adopting.’ (Expert 1) ‘To include an assessment of the capabilities customers currently have. If they need entirely new skills or infrastructure, it’s important to address these gaps beforehand.’ (Expert 2)	External change		

First, regarding recommendation on the design of the CMS and in relation to CMS robust value proposition, experts emphasize the importance of designing a clear and compelling business case that highlights the tangible benefits of CMS. This includes a detailed ROI analysis supported by quantitative financial data. In addition, the establishment of a structured, iterative approach to CMS design is recommended to align CMS offerings with both strategic objectives and operational needs of customers. This methodology must constitute a step-by-step progression through iterative stages of development, ensuring that services remain relevant and effective to customers. This iterative testing process, supported by principles from agile micro-service development, ensures adaptability and reliability in final deployments ( Iriarte *et al.*, 2023; Kamp *et al.*, 2023; Sjödin *et al.*, 2020).

For instance, experts encourage concepts from adaptive service design frameworks suggest integrating customer feedback loops using advanced digital tools to enhance alignment with operational contexts ( Iriarte *et al.*, 2023; Marcon *et al.*, 2022). Experts advocate for a deep understanding of customer requirement, actively involving customers in every stage of CMS development. In this sense, engaging with customers' environments through co-creation processes ( Iriarte *et al.*, 2023) is essential as identified in servitization literature emphasizing the value of co-developing solutions with key stakeholders to create tailored, effective solutions ( Marcon *et al.*, 2022; Raddats *et al.*, 2022).

In this sense, in addition to data analytics experts recommend the use of ethnographic practices for immersing in customer operations to understand strategic and operational contexts of customers by identifying and involving the right stakeholders throughout the customer's lifecycle. This includes observations to decision-makers, IT personnel, and technicians who directly interact with the service. Engaging a comprehensive network of stakeholders ensures that all perspectives are considered during service development and prototyping phases. Such involvement ensures the integration of diverse expertise and perspectives, aligning with recommendations in multi-actor service ecosystems where stakeholder collaboration drives effective solution design ( Iriarte *et al.*, 2023; Raddats *et al.*, 2022). Literature also highlights that this network approach can improve cross-functional communication and reduce barriers to implementation ( Ahmad & Kamaruddin, 2012; Gebauer *et al.*, 2005). This is also as identified in the literature on human-centred design and advanced service models ( Iriarte *et al.*, 2023; Nguyen *et al.*, 2022a; Nguyen *et al.*, 2022b; Sjödin *et al.*, 2020). Additionally, experts recommend conducting research to determine if the service addresses a market gap or introduces a new concept, and then shaping the business model accordingly. Collaborating with market associations is seen as a valuable strategy for gathering insights into customer behaviour and emerging trends. Finally, testing solutions through small-scale pilot projects before full-scale implementation is considered crucial by the experts. This iterative testing process helps to refine offerings, ensuring alignment with customer needs and minimize resistance to large-scale changes. According to the interviews, this iterative testing process will ensure adaptability and reliability in final deployments ( Kamp *et al.*, 2023; Zhang & Banerji, 2017).

**Figure 1.**
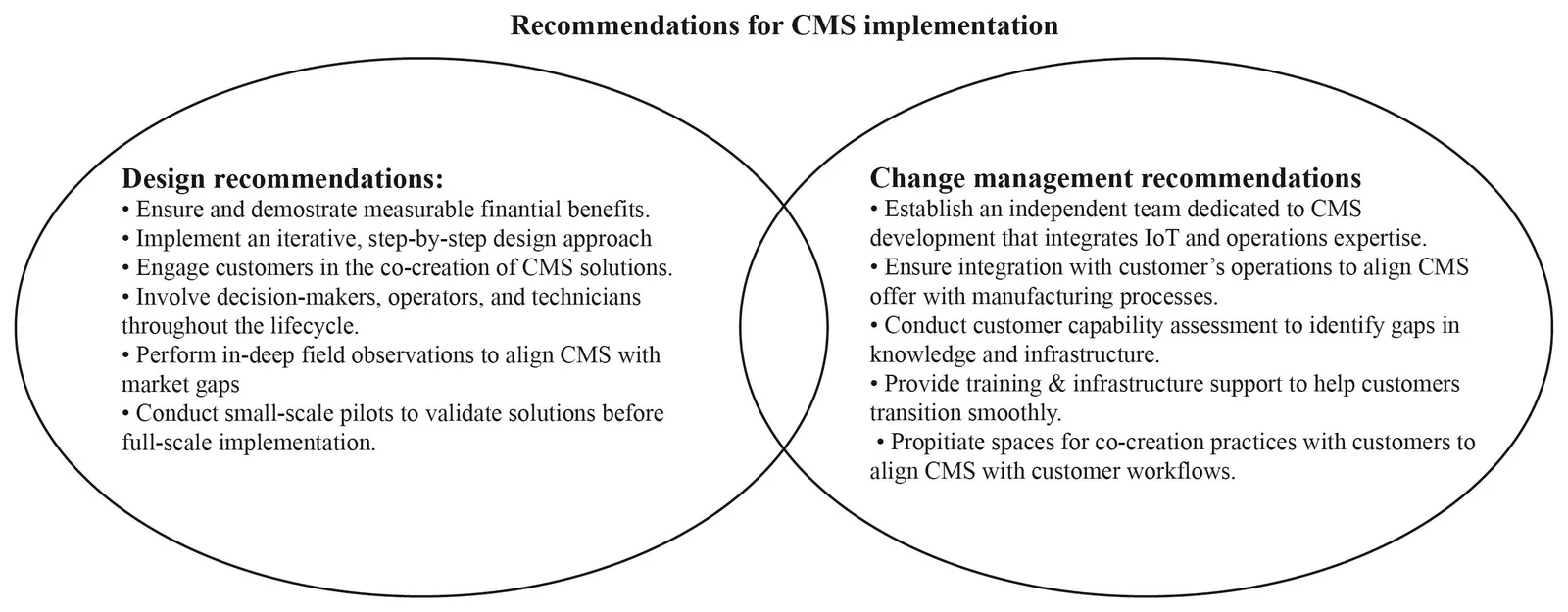
Visual representation of recommendations given by the experts to overcome CMS implementation challenges.

Second, regarding change management, experts divide them into internal and external change management recommendations for implementing CMS. For internal changes, experts advocate OEMs to establish an independent, multidisciplinary department dedicated to CMS design and development. Such an approach aligns with findings in the servitization literature, which highlight the need for clear internal structures that support the transition to service-oriented models ( Kowalkowski *et al.*, 2017; Nguyen *et al.*, 2022b). This department should operate closely with production units to ensure integration and adaptability to customer operations. By maintaining a dedicated team, companies can foster specialized expertise, enhance innovation, and ensure a focused approach to developing and delivering CMS offerings effectively ( Jardine *et al.*, 2006; Raddats *et al.*, 2022). This multidisciplinary approach can also facilitate interdepartmental communication ( Visnjic *et al.*, 2017). In terms of external changes, experts underline the importance of thoroughly understanding the adjustments required from customers to adopt the CMS successfully. Again, this brings us to in-field customer research. This includes investigating internal routines, processes, and the extent of changes customers must undertake, as seen in the successful implementation of digital service frameworks ( Iriarte *et al.*, 2023; Sjödin *et al.*, 2020). Such changes might involve retraining personnel, upgrading infrastructure, or adjusting operational workflows. Experts recommend conducting detailed field research to gain insights into customers’ internal processes and routines. Additionally, they suggest including an assessment of the current capabilities within customer organizations to identify gaps in skills or infrastructure. In this sense, recent research highlights that leveraging co-creation practices during capability assessments fosters collaborative problem-solving and reduces customer hesitance, thereby enhancing adoption rates and long-term success ( Kamp *et al.*, 2023; Zhang & Banerji, 2017). Addressing these gaps proactively—whether through tailored training programs or infrastructure enhancements—can help minimize resistance and ensure a smoother transition to the new system. Figure 1 summarizes and visually represents the recommendations given by the experts interconnecting the elements necessary for the successful adoption and scaling of CMS in industrial applications.

## Conclusions, limitations and future research

By leveraging expert elicitation, this study has thoroughly examined the specific challenges encountered by OEMs in implementing CMS in digital servitization, and more notably, has proposed practical solutions and actionable recommendations to practitioners to address these challenges effectively. By doing so, this research sheds light on a specific application of digital servitization; CMS implementation, moving beyond the theoretical generalizations on advanced services presented in the existing literature.

Therefore, at the theoretical level, on one hand, this study contributes to the digital servitization literature by providing an in-depth examination of the implementation challenges of CMS for OEMs in digital servitization. Unlike prior research on CMS that predominantly focuses on the technological aspects, the study reinforces the significance of stakeholder engagement and co-creation in CMS implementation. Furthermore, on the other hand, it enriches digital servitization theories by emphasizing the disconnect between technological readiness and actual market success. The findings provide empirical validation for the CMS case of the existing theoretical frameworks bridging the gap between theoretical perspectives and suggest practical solutions, offering actionable design and management recommendations that enhance the applicability of CMS by OEMs within Industry 4.0 environments.

In particular to this, and referring to practical contributions, our findings advocate that OEMs must create and establish independent multidisciplinary service units with a wide autonomy and sufficient decision-making capacity. These unit must integrate IoT expertise with operational knowledge, able to perform in-field customer research and co-design practices to engage customers’ stakeholders to ensure CMS solutions are aligned with operational needs. These capabilities have been largely undervalued by digital servitization literature in favour of emphasizing only technological development capabilities. We defend that these units must be able to perform autonomously CMS design process and lead internal and external change management processes. These practices address organizational barriers and fosters collaboration, ensuring the scalability and success of CMS implementations.

Future research in this specific field could be oriented to tackle the inherent limitations of the expert elicitation methodology used in this study. Expanding the pool of experts across broader geographical regions and diverse industrial sectors could help to provide more non-context depending insights. Moreover, incorporating quantitative approaches alongside qualitative insights could validate the findings and help generalize the practical recommendations for OEMs. On the contrary, an alternative research path could rely on experts’ elicitation focused on specific sectors in digital servitization (e.g., tool machinery, energy, health equipment, etc.) to address more specific challenges and solutions for CMS in each sector. Finally, longitudinal studies tracking the implementation and impact of CMS over time would be particularly valuable, as they could uncover dynamic factors affecting CMS success.

## Ethics statement

This study was approved by the Research Ethics Committee of Mondragon Unibertsitatea under reference number IEB-20240909, on 9th September 2024. All research procedures adhered to the ethical standards set by the committee and complied with the Declaration of Helsinki and its subsequent amendments. Informed consent to participate was obtained in advance, primarily in written form via email. In some instances, verbal consent was obtained. This approach was reviewed and approved by the ethics committee, given the remote nature of the study. Verbal consent—recorded at the time of the interview—was considered both ethically appropriate and logistically practical, supporting participant accessibility and the protection of data privacy.

## Data Availability

All research data underlying the results of this article—including the call for participants, consent forms, interview guides, and fully manually anonymised interview transcripts—are available on Mendeley Data under the terms of the Creative Commons Attribution 4.0 International License (CC BY 4.0). The interviews were transcribed using Tactiq; no other software was used for data processing or analysis. Mendeley: **Underlying data. Overcoming challenges in implementing condition monitoring services: expert elicitation**, 10.17632/r8bxnpyhkj.1 ( Iriarte *et al.*, 2025a) This project contains the following underlying data: interview transcripts Data is available under the terms of the Creative Commons Attribution 4.0 International License (CC BY 4.0). Mendeley: **Extended data. Overcoming challenges in implementing condition monitoring services: expert elicitation,** 10.17632/2y63y6zb8s.1, ( Iriarte *et al.*, 2025b) This project contains the following underlying data: call for participants, consent forms, and interview guides Data is available under the terms of the Creative Commons Attribution 4.0 International License (CC BY 4.0).
