# Overcoming Challenges in Implementing Condition Monitoring Services: Recommendations from an Expert Elicitation Exploratory Study

**DOI:** 10.12688/openreseurope.20044.2

**Published:** 2026-08-04

**Authors:** Ion Iriarte, Hien Nguyen Ngoc, Ganix Lasa

**Affiliations:** 1 Design Innovation Center, Mondragon University Higher Polytechnic School, Arrasate, Basque Country, 20500, Spain

**Keywords:** Internet of Things, digital servitization, product-service systems, condition monitoring services, digital manufacturing.

## Abstract

**Background:**

Condition Monitoring Services (CMS) have emerged as an important digitally enabled service offering within manufacturing servitization, allowing Original Equipment Manufacturers (OEMs) to remotely monitor equipment, improve maintenance performance, and create new service-based revenue streams. Despite the technological maturity of condition monitoring technologies, many OEMs continue to struggle with the successful implementation of CMS. This study aims to identify the challenges limiting CMS implementation, the solutions used to overcome these challenges, and the recommendations that can support successful CMS implementation.

**Methods:**

An exploratory expert elicitation approach was adopted. Semi-structured interviews were conducted with 13 experts from OEMs, universities, research centres, and business consultancy organizations who possessed substantial experience in CMS implementation, digital servitization, and Industry 4.0. The interview data were analysed using inductive thematic analysis.

**Results:**

The findings reveal that the principal barriers to CMS implementation are socio-technical rather than technological. Three interrelated groups of challenges were identified: challenges related to CMS value propositions, challenges associated with CMS design processes, and challenges concerning organizational and customer readiness. To address these challenges, experts proposed solutions centred on value quantification, co-creative and iterative service design, and service-oriented organizational capability development. The study further identified recommendations emphasizing customer-value-driven CMS design, dedicated and legitimate service units, and proactive management of organizational change.

**Conclusions:**

Successful CMS implementation depends less on the sophistication of condition monitoring technologies and more on the ability of OEMs to align technological resources, organizational capabilities, customer requirements, and value creation mechanisms. By providing empirically grounded insights into the challenges, solutions, and recommendations associated with CMS implementation, this study extends both CMS and digital servitization literature and offers actionable CMS-specific guidance for OEMs seeking to transform condition monitoring technologies into sustainable service businesses.

## Introduction

Industry 4.0 has accelerated the digital transformation of manufacturing and created new opportunities for servitization, enabling Original Equipment Manufacturers (OEMs) to complement physical products with data-driven services ( Kalsoom et al., 2020; Kowalkowski et al., 2017). Through servitization, OEMs seek to move beyond transactional product sales and develop long-term customer relationships based on value creation across the equipment lifecycle. Digital technologies such as the Internet of Things (IoT), cloud computing, and advanced analytics have further strengthened this transition by enabling new forms of service provision and value co-creation between manufacturers and customers ( Rymaszewska et al., 2017; Kristoffersen et al., 2021). Consequently, digital servitization has become a central mechanism through which manufacturers pursue competitive advantage in Industry 4.0 ( Agostini et al., 2026; Momenzadeh et al., 2026).

Within this context, Condition Monitoring Services (CMS) have emerged as an important digitally enabled service offering within manufacturers’ servitization strategies. CMS enable OEMs to remotely monitor the condition and performance of equipment deployed at customer facilities through connected sensors, data acquisition systems, and analytical tools ( Slatter et al., 2021). By providing continuous insight into equipment health and operational behaviour, CMS can support predictive maintenance, reduce unplanned downtime, optimize resource utilization, and improve lifecycle performance ( Lanzilotti et al., 2022; Wu & Pi, 2023). Consequently, OEMs are increasingly offering CMS as a means of generating service-based revenue streams ( Baines & Lightfoot, 2013; Hien et al., 2022).

The growing adoption of CMS reflects a broader shift toward Product-Service Systems (PSS), where value is created through a combination of physical products and lifecycle services. OEMs are no longer expected solely to deliver reliable equipment but also to support customer outcomes through advanced services. CMS implementation therefore requires more than technological expertise. It also involves the development of organizational capabilities that enable OEMs to transform data into customer value ( Valtakoski, 2017; Keh et al., 2012). Recent studies further suggest that the success of digital servitization initiatives depends on how organizations align these capabilities to support value creation and capture ( Agostini et al., 2026; Karatzas et al., 2026).

Despite the recognized potential of CMS, many implementation initiatives struggle to achieve their intended outcomes ( Bousdekis et al., 2020; Hien et al., 2022; March & Scudder, 2019). Specific research in CMS has largely focused on technical challenges, including sensor selection, data availability, data quality, and predictive model accuracy ( Goyal & Pabla, 2015; Selcuk, 2017). More broadly, maintenance-related research has long been criticized for emphasizing technical and analytical solutions while devoting comparatively less attention to broader implementation challenges in industrial practice ( Fraser et al., 2015).

Emerging research in the wider field of digital servitization suggests that implementation barriers associated with digitally enabled services frequently extend beyond technical considerations ( Münch et al., 2022). Research in digital servitization has highlighted the importance of value proposition design, service-oriented organizational capability development, organizational readiness, resource allocation, customer collaboration, and service delivery mechanisms in determining the success of digitally enabled services ( Antikainen et al., 2018; Ingemarsdotter et al., 2021; Lee, 2020a, 2020b). Moreover, recent evidence suggests that digital servitization initiatives are frequently hindered by organizational, strategic, and managerial barriers that interact in complex ways and may even generate unintended negative consequences ( Rietdijk, 2026).

In particular, Münch et al. (2022) argue from a socio-technical systems perspective that successful digital servitization depends on the development of complementary technological, organizational, and human capabilities. Similarly, Kohtamäki et al. (2020) show that servitization initiatives are characterized by inherent organizational tensions, requiring firms to balance competing demands related to resources, value creation, customer relationships, and service delivery. More recent studies suggest that such tensions intensify in digital servitization environments ( Paiola & Grandinetti, 2026; Smania et al., 2026). Collectively, these findings suggest that CMS implementation should be understood as a socio-technical and organizational transformation process rather than merely a condition monitoring technology deployment challenge.

Digital servitization literature has examined challenges related to organizational transformation, capability development, customer engagement, and business model innovation ( Kristiansen & Aas, 2026; Paiola & Grandinetti, 2026; Smania et al., 2026). However, these studies typically address digitally enabled services at a general level and provide limited insights into the specific implementation challenges and potential solutions associated with CMS. CMS possess distinctive characteristics, including continuous access to operational equipment data, integration with customer maintenance processes, dependence on predictive analytics capabilities, concerns regarding data ownership and cybersecurity, and the need to demonstrate measurable operational and financial value through maintenance-related outcomes.

Consequently, there remains limited empirical understanding of how technological, organizational, managerial, and relational factors interact during CMS implementation and how OEMs can transform condition monitoring technologies into commercially viable service offerings. While existing CMS research has largely concentrated on technical aspects, providing valuable insights into how condition monitoring technologies function, the broader digital servitization literature often examines digitally enabled services at a relatively high level of abstraction and frequently treats them as a homogeneous class of service offerings. As a result, considerably less attention has been devoted to understanding how manufacturers implement CMS as service offerings within their digital servitization strategies, including the challenges associated with creating, delivering, and capturing value from these services.

To address this gap, this study investigates: (i) the challenges perceived by the experts in CMS implementation, (ii) the solutions to overcome them, and (iii) the recommendations proposed for OEMs seeking to implement CMS offerings. The study contributes to the CMS and digital servitization literature in three ways. First, it extends the understanding of CMS implementation by conceptualizing it as a socio-technical challenge rather than a predominantly technological one. Second, it provides empirical evidence of how digital servitization tensions and paradoxes emerge in CMS implementation and identifies mechanisms through which OEMs can address them. Third, it translates broad digital servitization socio-technical principles into CMS-specific implementation guidance for OEMs.

## Theoretical background

### A socio-technical perspective on CMS implementation within digital servitization

CMS are digitally enabled service offerings through which OEMs collect, analyse, and interpret operational data from equipment deployed at customer facilities in order to support maintenance, operational optimization, and decision-making activities ( Jardine et al., 2006; Ingemarsdotter et al., 2021). Enabled by Industry 4.0 technologies CMS allow OEMs to move beyond traditional equipment sales and provide lifecycle-oriented services based on continuous monitoring of equipment conditions ( Paschou et al., 2020; Rymaszewska et al., 2017). Rather than being a technological solution alone, CMS allow OEMs to offer advanced maintenance, support customer outcomes, and generate new revenues through digital technologies ( Wang et al., 2024). From a digital servitization perspective, CMS can be understood as a specific type of PSS in which value is co-created through the integration of physical products, IoT technologies, and service activities.

The implementation of CMS therefore involves more than the deployment of sensors and analytical tools. It requires OEMs to integrate digital technologies with service development processes, organizational structures, customer engagement mechanisms, and new value propositions and business model innovation activities. Consequently, CMS implementation can be conceptualized as the process through which OEMs develop, deliver, and scale digitally enabled service offerings capable of generating value for both providers and customers ( Agostini et al., 2026; Momenzadeh et al., 2026).

Although digital technologies enable new service opportunities for OEMs, successful implementation depends on the development of complementary organizational and managerial capabilities ( Momenzadeh et al., 2026). OEMs must redesign internal processes and structures, develop service-oriented cultures, acquire new organizational capabilities, establish data governance mechanisms, and collaborate closely with customers throughout service delivery ( Marcon et al., 2022). Consequently, CMS implementation should not be understood as a purely technological challenge but rather as an organizational transformation process within digital servitization affecting multiple dimensions ( Münch et al., 2022).

To better understand CMS implementation challenges, this study adopts a socio-technical perspective on digital servitization. According to socio-technical systems theory, organizational outcomes emerge from the interaction between technological systems and social systems, rather than from technology alone ( Münch et al., 2022). This perspective is particularly useful for CMS implementation because the value generated by condition monitoring technologies ultimately depends on how OEMs integrate technical solutions with customer operations, business processes, and service delivery mechanisms. Accordingly, implementation success may be constrained not only by technological limitations but also by organizational resistance, organizational capability gaps, weak value propositions, or difficulties in coordinating stakeholders. In addition, manufacturers frequently experience negative synergistic effects when multiple digital servitization barriers interact simultaneously, creating implementation difficulties that extend beyond isolated technological or organizational problems ( Rietdijk, 2026).

### Challenges in CMS implementation

Although condition monitoring technologies have matured considerably in recent years, the successful implementation of CMS remains a challenging undertaking for many OEMs. Increasingly, researchers argue that the principal barriers to digital servitization are not technological in nature but rather stem from organizational, managerial, and relational factors that hinder the transformation of technological capabilities into customer value ( Marcon et al., 2022). The CMS literature starts to reflect similar observations. While technical challenges such as data quality, component selection, and predictive modelling remain important ( Goyal & Pabla, 2015; Selcuk, 2017), a growing body of research points towards broader implementation difficulties related to customer collaboration, organizational readiness, organizational capabilities, change management, and business model development ( Lee, 2020a, 2020b; Valtakoski, 2017; Werbińska-Wojciechowska & Winiarska, 2023).

One recurring challenge concerns the development of strong value propositions and business models for digitally enabled services. While CMS can generate significant operational benefits, OEMs often struggle to translate these benefits into compelling customer value propositions and sustainable revenue mechanisms. Research on digital servitization consistently reports tensions between traditional product-oriented logics and service-oriented value creation approaches, particularly when firms attempt to monetize digital services and justify investments to customers ( Valtakoski, 2017; Visnjic et al., 2017; Kamp et al., 2023). These difficulties frequently contribute to the “service paradox”, in which manufacturers invest heavily in service development without obtaining the expected financial returns ( Gebauer et al., 2005; Kowalkowski et al., 2017). More recent research suggests that these tensions become particularly pronounced in digitally enabled services, where manufacturers must simultaneously balance digital technology investments, customer value creation, service monetization, and organizational transformation efforts ( Paiola & Grandinetti, 2026).

A second challenge concerns organizational transformation and organizational capability development. The implementation of CMS frequently requires the creation of new roles, skills, routines, and service-oriented organizational structures that differ substantially from those of product-centric manufacturing organizations. Employees must develop service-oriented competences, data interpretation capabilities, and collaborative working practices that enable the effective delivery of digital services ( Münch et al., 2022). In addition, manufacturers often face difficulties reallocating resources, coordinating cross-functional teams, and aligning service activities with existing operational structures ( Marcon et al., 2022). These organizational challenges help explain why technologically mature condition monitoring solutions frequently fail to achieve widespread adoption.

A third challenge relates to customer collaboration and stakeholder alignment. Digital servitization requires deeper interactions between OEMs and customers because value is co-created through the continuous use of data, operational knowledge, and service expertise ( Kohtamäki et al., 2019). However, manufacturers frequently encounter resistance regarding data sharing, concerns about data ownership, lack of trust, and uncertainty surrounding responsibilities and risks associated with digital services ( Ingemarsdotter et al., 2021; van de Kerkhof et al., 2016). These challenges are particularly significant in CMS because access to operational data is a prerequisite for CMS delivery. As a result, successful implementation depends on establishing collaborative relationships and governance mechanisms that facilitate information exchange and mutual trust. Such collaborative arrangements are increasingly important in digital servitization environments, where value is co-created across organizational boundaries and depends on the active participation of multiple stakeholders throughout the service lifecycle ( Paiola & Grandinetti, 2026; Smania et al., 2026).

### Approaches to successful CMS implementation

Beyond identifying challenges, recent literature in digital servitization has suggested some possible approaches that support successful CMS implementation. A recurring argument is that implementation success depends on the simultaneous development of technological, organizational, and human capabilities ( Münch et al., 2022). Consequently, investments in IoT infrastructures and analytics should be accompanied by investments in workforce development, service management competences, customer engagement, and organizational structures capable of supporting service delivery.

Several studies have shown that many digital service initiatives in manufacturing fail because firms are unable to capture an appropriate share of the value they create ( Kamp et al., 2023). These difficulties are increasingly recognized as critical barriers to realizing the performance benefits of digital servitization, particularly when firms struggle to align value creation mechanisms with business model innovation and service monetization strategies ( Agostini et al., 2026; Karatzas et al., 2026). Linde et al. (2023) argue that successful digital servitization requires the development of suitable revenue models that align value creation with value capture throughout the service lifecycle. Examples include subscription models, outcome-based contracts, pay-per-use arrangements, and performance-based agreements. Consequently, the successful implementation of CMS requires not only technical feasibility but also mechanisms that enable sustainable monetization of the generated value.

Several authors highlight the importance of organizational transformation during digital servitization. Münch et al. (2022) argue that successful digital servitization requires the development and alignment of technological, organizational, and human capabilities, while Marcon et al. (2022) emphasize that manufacturers must redesign structures, processes, and organizational practices to support digitally enabled service offerings. Similarly, Zhang et al. (2025) demonstrate that successful digital servitization implementation is closely associated with organizational change processes involving culture, learning, organizational capability development, and strategic alignment. These studies suggest that OEMs must actively manage change rather than treating CMS implementation as a standalone technological project. Similarly, Minaya et al. (2026) show that managers’ strategic perceptions of Industry 4.0 significantly influence the extent to which firms embrace digital servitization initiatives and redesign their organizations around service-oriented objectives.

Recent research further suggests that manufacturers are often required to simultaneously pursue competing goals such as standardization and customization, efficiency and flexibility, technological innovation and customer intimacy, as well as exploration of new service opportunities and exploitation of existing product businesses ( Paiola & Grandinetti, 2026; Smania et al., 2026). Rather than eliminating these tensions, literature suggests that successful organizations develop organizational mechanisms and coping practices that enable them to coexist with these tensions over time. This implies that increasing service complexity requires organizational structures, governance mechanisms, and managerial practices capable of balancing competing demands related to customer value creation, operational efficiency, standardization, and customization ( Kohtamäki et al., 2020; Paiola & Grandinetti, 2026).

Leadership also plays an important role in the implementation process. Tagscherer and Carbon (2023) highlights that digital servitization requires leaders capable of articulating a service-oriented vision, coordinating multidisciplinary teams, fostering organizational learning, and managing resistance to change. Leadership therefore becomes a key mechanism for aligning technological initiatives with strategic objectives and facilitating the transition from product-centric to service-oriented business models.

Finally, successful implementation of CMS often relies on iterative and collaborative approaches that actively involve customers throughout service development and deployment. Pilot projects, co-creation activities, gradual service extensions, and continuous feedback mechanisms have been identified as effective practices for reducing uncertainty, validating value propositions, and building trust among stakeholders ( Sjödin et al., 2020; Iriarte et al., 2023). These approaches enable firms to refine service offerings while simultaneously increasing customer readiness, stakeholder commitment, and organizational learning. Such practices are particularly relevant in CMS, where service success depends on continuous interaction between OEMs and customers and on the shared use of operational data.

### Research gap

Despite the growing body of research on digital servitization, current knowledge regarding how to overcome the specific CMS implementation challenges remains fragmented. Existing studies typically address isolated challenges of implementation, such as technical solutions for data acquisition and predictive analytics ( Lee et al., 2014), organizational capabilities for digital servitization ( Münch et al., 2022), digital servitization business models and value capture mechanisms ( Agostini et al., 2026), service revenue mechanisms ( Linde et al., 2023), or customer co-creation practices in servitization ( Sjödin et al., 2020; Iriarte et al., 2023).

While these contributions provide valuable insights, they are often examined independently and therefore offer a partial view of the implementation process. As a result, knowledge about how these different dimensions interact in practice remains limited. This limitation is particularly important because recent research increasingly portrays digital servitization as a multidimensional socio-technical transformation process involving organizational capabilities, organizational legitimacy, stakeholder relationships, value proposition and business models, and organizational tensions and paradox management ( Kristiansen & Aas, 2026; Paiola & Grandinetti, 2026; Momenzadeh et al., 2026).

In addition, much of the existing literature in digital servitization discusses implementation challenges and solutions at a relatively high level of abstraction and treats digital services in a comparatively homogeneous manner. Recommendations frequently focus on broad organizational capabilities, digital transformation strategies, or business model adaptation ( Kohtamäki et al., 2020; Tagscherer & Carbon, 2023; Zhang et al., 2025). Although these recommendations are managerially valuable, they are often developed for digital servitization in general and are not specifically linked to the particular implementation conditions of CMS. CMS present unique characteristics that distinguish them from many other digital services, including continuous access to operational equipment data, integration with customer maintenance processes, management of data ownership and cybersecurity concerns, dependence on predictive analytics capabilities, and the need to demonstrate measurable operational and financial value from maintenance-related outcomes. Consequently, generic digital servitization recommendations may not fully address the practical realities encountered by OEMs in implementing CMS.

Therefore, there remains a need for more empirical research capable of providing a grounded understanding of CMS implementation. By focusing on expert perspectives, this study seeks to generate actionable and context-sensitive insights that complement existing theoretical knowledge and provide a more specific understanding of CMS implementation in OEM environments.

Ketokivi and Choi (2014) indicate that exploratory qualitative studies are particularly valuable when existing knowledge remains fragmented and when theory development benefits from understanding complex real-world phenomena through expert perspectives. Accordingly, this study adopts an exploratory expert elicitation approach to investigate CMS implementation by OEMs. In particular, the study addresses the following research questions:

• RQ1: What are the implementation challenges that limit market success of CMS?
• RQ2: What are the potential solutions to overcome these challenges?
• RQ3: What recommendation should an OEM take to offer CMS successfully?

## Methodology

### Research approach

This study adopts an exploratory qualitative research design based on expert elicitation. The approach is appropriate because CMS implementation by OEMs represents a complex socio-technical phenomenon involving technological, organizational, managerial, and customer-related dimensions whose interactions remain insufficiently understood in the existing literature.. Although prior research in CMS implementation and digital servitization literature has identified a variety of technical and organizational barriers, current knowledge remains fragmented and provides limited understanding of how these challenges interact during real-world CMS implementation. Furthermore, existing solutions within digital servitization literature for successful CMS implementation tend to be formulated at a relatively generic level and are often insufficiently connected to the specific characteristics of CMS. Consequently, there is a need for context-sensitive and practice-oriented insights capable of complementing existing theoretical knowledge and improving understanding of CMS implementation by OEMs.

Expert elicitation is particularly suitable when investigating highly specialized domains where relevant knowledge is dispersed among experienced practitioners and researchers and when the objective is to obtain context-rich insights rather than statistical generalization ( Bogner & Menz, 2009; Gläser & Laudel, 2009; Meuser & Nagel, 2009). In our context, CMS implementation requires expertise spanning CMS implementation, advanced services, digital servitization, and Industry 4.0. Consequently, expert interviews provide access to practical knowledge and implementation experiences that cannot be easily captured through surveys or quantitative approaches.

The study follows an exploratory logic consistent with Ketokivi and Choi (2014), who argue that qualitative inquiry is particularly valuable when existing theory does not sufficiently explain a complex managerial phenomenon and when deeper contextual understanding is required. The objective is therefore not statistical generalization but the generation of analytically generalizable insights regarding the challenges, solutions, and recommendations associated with CMS implementation by OEMs.

### Expert selection and sampling

Following Gläser and Laudel (2009), expertise was defined as possessing specialized knowledge and practical experience directly relevant to CMS implementation. Experts were selected using purposive sampling because the objective was to access information-rich participants capable of providing informed perspectives on the phenomenon under investigation.

To be included in the study, participants had to meet the following criteria: (i) direct involvement in CMS design, development, deployment, or management; and (ii) demonstrated expertise in one or more relevant domains, including digital servitization, advanced services, industrial service design, industrial service management, or Industry 4.0.

Given the multidisciplinary nature of CMS implementation, particular attention was devoted to incorporating complementary perspectives spanning technological, organizational, and business domains. CMS implementation requires not only expertise in monitoring technologies and data analytics, but also knowledge of servitization, service development, customer value creation, and business model innovation. Therefore, experts were intentionally selected from different but complementary backgrounds, including OEMs, business consultancy, universities, and industrial research centres. This strategy was adopted to capture the multidimensional nature of CMS implementation and reduce the risk of relying on a single organizational perspective.

A list of 20 potential experts meeting the selection criteria described above was compiled. Experts were identified through professional networks, previous research collaborations, industrial projects, and conference participation. Thirteen experts accepted the invitation to participate and were included in the study ( Table 1).

**Table 1.** Expert profile.

Expert	Major fields	Business	Role	Working years	Background and relevance
Expert #1	Software and Systems Engineering	University	Researcher	18	Contributing knowledge on digital infrastructures, system integration, and data management capabilities required for CMS implementation.
Expert #2	Service Design, New Service Development	University	Researcher	11	Providing expertise on service-oriented value proposition design, customer involvement, and service innovation processes relevant to CMS implementation
Expert #3	Servitization, PSS, Advanced Services	Business Consultancy	Senior Consultant	22	Senior consultant supporting manufacturers in servitization transition, contributing practical experience on business model transformation and organizational change.
Expert #4	Servitization, Business Model Innovation	University	Researcher	19	Providing expertise on value capture, service strategy, and organizational transformation associated with advanced services.
Expert #5	Digital Manufacturing, IoT, Automation	University	Researcher	11	Researcher specialized in Industry 4.0 technologies, IoT, and automation, contributing knowledge on technological enablers required for CMS implementation.
Expert #6	Servitization, PSS, Advanced Services	Research Centre	Researcher	23	Applied researcher in a machine-tool, involved in industrial servitization and advanced maintenance projects with OEMs, providing extensive experience in CMS implementation across multiple firms.
Expert #7	Service Management, Service Operations	OEM	Service Manager	9	Service manager directly responsible for industrial service delivery, contributing first-hand experience regarding customer adoption, service operations, and CMS commercialization.
Expert #8	New Service Development, IoT, Automation	OEM	Head of IoT and Automation	21	Industrial manager leading IoT-enabled service initiatives and digital transformation projects, providing practical insights into CMS deployment, data integration, and organizational change.
Expert #9	New Service Development, IoT, Automation	OEM	Researcher in IoT and Automation	26	Senior industrial expert involved in the development of IoT-based solutions and advanced maintenance services, contributing extensive experience in the technological and operational aspects of CMS implementation.
Expert #10	Servitization, PSS, Advanced Services	Research Centre	Head of R&D	23	Head of R&D in a machine-tool technology research centre, leading collaborative projects with OEMs on servitization, advanced services, and digital manufacturing solutions.
Expert #11	Software R&D, Servitization, PSS	Research Centre	Head of RDI Software Development	14	R&D manager responsible for software development and digital service innovation projects, contributing expertise on software-enabled servitization and industrial digital platforms.
Expert #12	Service Management, Service Operations	OEM	Service Manager	11	Service manager with direct responsibility for industrial service operations and customer support activities. Contributes practical insights into the delivery of advanced services, customer readiness, and the organizational challenges associated with implementing CMS.
Expert #13	Service Design, New Service Development	Research Centre	Development Manager	20	Development manager in a machine-tool technology research centre, responsible for new service development initiatives conducted in collaboration with OEMs. Contributes expertise on translating technological capabilities into customer-oriented service offerings, and designing advanced service concepts.

The final sample consisted of 13 experts from Spain, Portugal, the United Kingdom, Sweden, Finland, and Germany. Four experts were employed by OEMs in roles directly related to CMS operations. One expert was a senior consultant specialized in manufacturing servitization. Four experts were researchers from universities with recognized expertise in servitization, industrial service design, Industry 4.0, and digital manufacturing. The remaining four experts belonged to several research centres specialized in machine-tool equipment and advanced manufacturing systems. These last four experts held senior research positions and possessed extensive experience participating in industrial projects focused on machine monitoring, IoT-enabled services, advanced maintenance solutions involving OEMs.

The sampling strategy intentionally combined experts from industrial practice and applied research environments to ensure a multidimensional understanding of CMS implementation and to avoid relying on a single organizational perspective. As CMS implementation involves technological, organizational, and business challenges that frequently transcend the boundaries of individual firms, research centres experts were considered particularly valuable because of their broad exposure to multiple OEMs. Their contributions complemented the direct operational insights provided by OEMs participants and enabled a richer understanding of CMS implementation challenges and solutions.

In total, the experts possessed between 9 and 26 years of professional experience, with an average of approximately 18 years. This combination of practical industrial knowledge, applied research expertise, and servitization consultancy experience provided a robust basis for exploring implementation challenges, potential solutions, and recommendations associated with CMS implementation by OEMs.

### Data collection

Data were collected through semi-structured expert interviews, a method that provides the flexibility required to explore complex and emerging phenomena ( Miles et al., 2013). Given the exploratory nature of the study and the objective of obtaining practical insights into CMS implementation, semi-structured interviews were considered particularly appropriate because they allow experts to draw upon their own experiences while enabling the researchers to probe relevant implementation issues in greater depth ( Bogner & Menz, 2009; Meuser & Nagel, 2009).

Prior to each interview, participants received an information package containing the research objectives, a brief summary of the study background, confidentiality assurances, and an informed consent statement. Following the recommendations of Saunders et al. (2016), this preliminary information helped participants understand the purpose and scope of the study while enhancing the credibility and transparency of the interview process. Participants were informed that their identities would remain confidential and that the collected material would be used exclusively for research purposes. Consent was obtained through signed consent forms and/or verbal confirmation before the interviews were recorded.

An interview guide was developed to ensure consistency across interviews while maintaining sufficient flexibility to explore emerging themes. The complete interview protocol including the interview guide is available in the Extended Data repository ( Iriarte et al., 2026). The interview guide was structured around the three research questions of the study and focused on: (i) Challenges associated with CMS implementation, (ii) Solutions for overcoming these challenges, and (iii) Recommendations for achieving successful CMS implementation.

To minimize response bias ( Myers & Newman, 2007), interviews began with broad, open-ended questions inviting experts to freely discuss their experiences and perspectives regarding CMS implementation. Once experts had provided their initial reflections, follow-up questions were used to explore specific issues in greater depth, including technological, organizational, managerial, customer-related, and business model aspects of implementation. This approach allowed experts to introduce themes that they considered relevant while ensuring adequate coverage of the research questions.

The semi-structured format also provided interviewers with the flexibility to adapt the sequence and wording of questions according to each participant’s expertise and professional background. Following the recommendations of Cenamor et al. (2017) and Raddats et al. (2022), emerging topics were explored whenever they contributed to a richer understanding of the phenomenon under investigation. This iterative and conversational format was particularly valuable given the multidisciplinary nature of CMS implementation and the varied backgrounds of the participating experts.

To further reduce potential social influence and reciprocal bias, each expert was interviewed individually rather than in group settings, following the recommendations of Calabrese et al. (2019). All interviews were conducted by two authors to maintain consistency in the interview process and interpretation of responses ( Benedettini, 2022).

The interviews were conducted in English between October and November 2024 and lasted between 40 and 60 minutes, with an average duration of approximately 50 minutes. All interviews were audio-recorded and transcribed for subsequent analysis. The resulting dataset comprised approximately 90 pages of interview transcripts, representing a rich source of expert knowledge regarding the challenges, solutions, and implementation recommendations associated with CMS deployment by OEMs.

### Data analysis

The interview data were analysed using an inductive thematic analysis approach ( Braun & Clarke, 2006; Thomas, 2006). This approach was selected because the objective of the study was to explore how experts perceive the challenges, solutions, and recommendations associated with CMS implementation without imposing predefined theoretical categories. Consistent with the exploratory nature of the research, the analysis sought to allow relevant patterns and themes to emerge from the empirical material while remaining guided by the study’s research questions.

The analysis was conducted manually by two researchers following an iterative five-stage process of independent coding, analytical discussion, thematic refinement, and consensus building. First, all interview transcripts were reviewed multiple times to achieve familiarity with the data and obtain a holistic understanding of the experts’ experiences and viewpoints. During this phase, researchers recorded preliminary observations, recurring challenges, proposed solutions, and implementation practices identified across the interviews.

Second, meaningful statements related to the three research questions were extracted from the transcripts and assigned initial descriptive codes. A meaningful statement was defined as any segment of text containing relevant information regarding implementation challenges, solutions, or recommendations for successful CMS implementation. This initial coding generated a large set of data-driven codes grounded directly in the experts’ narratives.

Third, the initial codes were compared, grouped, and organized into preliminary categories according to their conceptual similarity. This process involved continuously comparing codes across interviews to identify recurring patterns as well as divergences in expert perspectives. During this stage, categories were iteratively refined through discussions between the researchers to ensure a consistent interpretation of the data.

Fourth, preliminary categories were further reviewed, merged, refined, and consolidated into first-order themes. This process required the elimination of overlapping categories and the clarification of boundaries between themes. The purpose was to ensure that each first-order theme represented a coherent and distinct implementation issue discussed by the experts.

Finally, related first-order themes were aggregated into broader second-order themes representing higher-level patterns across the dataset. These second-order themes constituted the analytical structure used to present the findings and provided an integrative view of the challenges, solutions, and recommendations associated with CMS implementation. The final thematic structure subsequently informed the organization of the findings around challenges, solutions, and recommendations corresponding to the three research questions.

Following this procedure, the analysis progressed from raw interview data to increasingly abstract levels of interpretation. Across the 13 interviews, a total of 177 meaningful statements were identified and coded. These statements were subsequently grouped into 30 preliminary categories, which were refined into 25 first-order themes and ultimately consolidated into eight second-order themes. Table 2 summarizes the progression of the analysis process.

**Table 2.** Summary of the data analysis process.

Analytical process	Description	Output
Familiarization	Repeated reading of 13 interview transcripts and recording of initial observations	Understanding of dataset
Initial coding	Identification of meaningful statements related to the research questions	177 meaningful statements
Category development	Grouping of similar codes into preliminary categories	30 categories
Theme refinement	Merging and refining categories to eliminate redundancy	25 first-order themes
Theme aggregation	Grouping first-order themes into higher-level conceptual themes	8 second-order themes

To enhance analytical rigor, both researchers independently reviewed the transcripts and participated in regular analytical discussions throughout the coding process. Rather than measuring statistical inter-coder reliability, the study followed a reflexive thematic analysis approach in which differences in interpretation were actively discussed and used to refine theme development until a shared understanding of the data was achieved ( Braun & Clarke, 2006). This collaborative process strengthened the credibility, consistency, and transparency of the analysis.

### Data saturation

Given the exploratory nature of the study and the use of expert elicitation, the adequacy of the sample was assessed through thematic saturation rather than statistical representativeness ( Guest et al., 2006; Mason, 2010). Thematic saturation refers to the point at which additional interviews cease to generate substantially new concepts, themes, or insights relevant to the research questions. Consequently, the objective was not to reach a predetermined sample size but to continue data collection until the identified themes became sufficiently stable and recurring across participants.

To assess saturation, data collection and analysis were conducted concurrently. Following the recommendations of Guest et al. (2006), interviews were analysed sequentially in successive rounds, allowing the researchers to monitor the emergence of new insights throughout the research process. As coding progressed, attention was paid to the number of new meaningful statements and first-order themes generated in each analytical round.

As shown in Table 3, the first six interviews generated the majority of the implementation challenges, solutions, and recommendations identified in the study. The subsequent four interviews expanded the thematic coverage of the dataset and provided additional contextual variation. During the final analytical round, the last three interviews contributed further examples and supporting evidence but did not result in the identification of new first-order themes. Instead, these interviews reinforced and enriched the existing thematic structure. This pattern indicated that additional data collection was unlikely to substantially modify the analytical framework and therefore thematic saturation was considered achieved.

**Table 3.** Assessment of thematic saturation.

Analytical round	Interviews analysed	New statements	Emerging first-order themes	Emerging second-order themes	Saturation notes
Round 1	Experts 1–6	102	17	6	Majority of implementation challenges, solutions, and recommendations identified
Round 2	Experts 7–10	48	8	2	Additional variation and contextual insights identified
Round 3	Experts 11–13	27	0	0	No substantially new themes emerged; interviews provided confirmation and additional examples
Final output	13 experts	177	25	8	Thematic saturation achieved

This iterative process enhanced the credibility and dependability of the findings while ensuring that the resulting themes remained firmly grounded in the empirical material. Together, the expert selection procedure, the systematic coding process, the achievement of thematic saturation, and the transparent reporting of the analytical steps strengthen the credibility, dependability, and analytical robustness of the study’s findings.

## Findings: Challenges, solutions, and recommendations for CMS implementation

The analysis identified findings corresponding to the three research questions of the study. First, experts described the challenges that limit the successful implementation of CMS. Second, they proposed practical solutions for overcoming these challenges. Finally, they offered recommendations for OEMs seeking to implement CMS successfully.

The detailed coding structure, including first-order themes, second-order themes, and illustrative quotations, is available in the Extended Data (Tables E1 to E4). The findings presented below focus on the higher-level themes that emerged from the analysis.

### Challenges in CMS implementation

The analysis identified three main categories of implementation challenges that hinder the successful implementation of CMS: (i) challenges related to the CMS value proposition, (ii) challenges related to the CMS design process, and (iii) challenges related to organizational and customer readiness. Together, these themes answer RQ1.

***Challenges related to the CMS value proposition***

One of the most prominent challenges identified by the experts concerned the development and communication of the CMS value proposition. According to the interviewees, many CMS initiatives fail because customers do not perceive sufficient value to justify the investments, organizational changes, and risks associated with CMS adoption.

The interviews consistently indicated that OEMs struggle to demonstrate to their customers how CMS create value beyond existing maintenance practices. Although technical benefits may be evident, customers frequently fail to recognize how these benefits translate into meaningful operational or financial outcomes. As a result, the perceived benefits of CMS are often insufficient to motivate customers to modify existing practices and trust in CMS offerings.

A further challenge identified by the experts is the lack of value quantification in the value proposition. Many OEMs struggle to express CMS benefits through measurable indicators such as financial savings, productivity improvements, asset availability, or downtime reduction. Without tangible evidence of economic value, customers frequently find it difficult to justify investments in CMS and compare them against alternative priorities competing for organizational resources.

Another challenge concerns the difficulty of developing value propositions that address multiple customer stakeholder groups simultaneously. Experts observed that CMS offerings are often designed from a technical perspective without adequately considering the different expectations of managers, maintenance personnel, operators and other actors involved in the customer’s organization. Consequently, a value proposition that appeals to one stakeholder group may not necessarily be perceived as valuable by others.

***Challenges related to the CMS design process***

The second group of challenges relates to how CMS offerings are designed conceptually and developed for deployment.

First, experts highlighted difficulties of OEMs in understanding customer service-requirements. Several participants noted that OEMs frequently rely on assumptions regarding customer needs rather than systematically validating them through customer engagement activities. This often results in CMS offerings that fail to reflect the operational realities of different customer contexts.

Second, customer heterogeneity was considered a particularly important issue. According to the experts, potential customers differ significantly in terms of technological maturity, maintenance capabilities, organizational culture, resources, and willingness to adopt new CMS. However, CMS are frequently developed using generic product-centric design approaches that assume similar conditions across customers. Such assumptions can result in misalignments between CMS characteristics and customer capabilities.

Another challenge concerns access to customer resources. Successful CMS implementation often requires access to operational data, equipment, facilities, processes, and personnel. Experts reported that customers are frequently reluctant to provide such access due to concerns related to confidentiality, trust, cybersecurity, data ownership, and operational risk. Consequently, obtaining access to the resources required for CMS delivery represents a significant obstacle during implementation.

***Challenges related to CMS requirements and customer readiness***

The third challenge category concerns the organizational and operational requirements necessary to support CMS implementation, both by OEMs and their customers.

Experts observed that CMS implementation frequently requires changes in infrastructures, systems, processes, competences, and organizational routines, both in OEMs and customers. Difficulties arise when CMS must be integrated into existing operational environments that were not originally designed to support digital services. Legacy equipment, incompatible systems, and fragmented organizational processes were repeatedly cited as major barriers.

Lack of organizational readiness was another recurring concern. Interviewees noted that many OEMs attempt to develop and implement CMS without developing the skills, service capabilities, and structures necessary to support advanced service delivery. Existing personnel often possess strong technical competences but limited experience in service development, customer engagement, financial analysis, and value-based selling.

Finally, experts emphasized customer readiness challenges. In many cases, customers are hesitant to modify established routines, working practices, and decision-making processes. According to the interviewees, implementation difficulties frequently arise not because of customers’ technological limitations but because customers perceive the required organizational and behavioural changes as too demanding. Consequently, customer willingness to change becomes a critical factor influencing CMS adoption.

In response to RQ1, the study demonstrates that CMS implementation challenges extend well beyond technological considerations. The findings reveal three interconnected groups of barriers: challenges related to CMS value proposition, challenges associated with CMS design process, and challenges concerning organizational and customer readiness. Collectively, these challenges suggest that the market success of CMS is largely determined by how effectively OEMs create and communicate value, align service offerings with customer requirements, and manage the organizational changes required on both OEM and customer sides. Therefore, the major barriers to CMS implementation are rooted less in the maturity of condition monitoring technologies and more in the socio-technical challenges associated with value creation, service development, organizational transformation, and customer alignment.

### Solutions to CMS implementation challenges

The experts proposed a range of solutions to overcome the implementation challenges identified above. Consistent with the challenge structure, the proposed solutions can be grouped into three categories: solutions related to the CMS value proposition, solutions related to the CMS design process, and solutions related to organizational and customer readiness.

### Solutions related to the CMS value proposition

To address value proposition challenges, experts consistently emphasized the importance of developing robust and clearly articulated CMS value propositions.

A first solution involves demonstrating value through tangible benefits and measurable outcomes. According to the interviewees, CMS offerings should clearly communicate operational improvements and economic benefits rather than focusing primarily on technical features and functionalities. Customers need to understand how the CMS contributes to their business performance and operational objectives.

Therefore, experts repeatedly argued that CMS value propositions should be supported by clear metrics, financial indicators, and business cases. Quantifiable evidence related to productivity improvements, downtime reduction, maintenance savings, and return on investment was considered essential for reducing uncertainty and facilitating adoption.

Experts also stressed the importance of adopting a broader perspective on value creation by considering multiple stakeholder groups within customer organizations. Rather than designing CMS exclusively for maintenance departments, OEMs should consider the needs of managers, operators, IT technicians, and other stakeholders within customer organizations involved in decision-making and service use.

Finally, several interviewees advocated modular service offerings. Rather than introducing comprehensive CMS packages from the outset, OEMs should provide smaller and lower-risk service modules that allow customers to progressively experience and validate the benefits of CMS before expanding adoption towards more advanced service levels. In this sense, another commonly proposed solution was to target innovative customers and early adopters during the initial stages of implementation. According to the experts, pilot customers provide valuable learning opportunities while simultaneously generating evidence of service value that can later support wider adoption among more conservative customer groups.

***Solutions related to the CMS design process***

Regarding CMS design processes, experts highlighted the importance of adopting more co-creative and iterative development approaches.

A recurring recommendation was the use of co-creative and iterative service design processes that continuously incorporate customer feedback and learning during CMS development and beyond. According to the interviewees, CMS should evolve through repeated cycles of testing, feedback, refinement, and validation rather than through linear design approaches.

In addition, experts emphasized the need for more rigorous customer segmentation activities. OEMs should systematically investigate customer requirements, operational realities, and levels of technological and organizational readiness before developing their CMS offerings. In this sense, several participants highlighted the importance of on-site observation in customers’ facilities and collaborating with industry associations, customer networks, and other ecosystem actors to improve understanding of customer needs and market dynamics.

Finally, participants highlighted the need to address concerns regarding access to customer resources and data. Building trust through co-creation practices, transparency, clear communication, and appropriate governance mechanisms was considered essential for facilitating customer adoption of CMS offerings.

***Solutions related to organizational and customer readiness***

The third group of solutions focused on increasing readiness for CMS implementation within both OEM and customer organizations.

To improve integrability, experts proposed gradual implementation strategies based on modular services, pilot projects, and phased deployments. Such approaches allow organizations to experiment with CMS on a smaller scale before committing to broader organizational changes. This is related to solutions for CMS design processes.

Service-oriented organizational capability development emerged as another important solution. Experts emphasized the need to strengthen competences related to service management, customer collaboration, business development, financial analysis, and value communication within dedicated service units. According to the experts, many OEMs possess strong technical and engineering capabilities but lack the service-oriented skills required to effectively commercialize and scale CMS offerings. In particular, organizations need capabilities to identify customer needs, translate technical functionalities into customer value propositions, quantify business benefits, develop sustainable revenue models, and manage long-term customer relationships.

Experts also highlighted the importance of multidisciplinary teams capable of integrating expertise from engineering, digitalization, maintenance, sales, marketing, and service management. Such capabilities enable organizations to bridge the gap between technological development and market adoption, improve internal coordination, and support more effective value creation, delivery, and capture. Consequently, service-oriented organizational capability development was viewed not only as a means of improving CMS implementation but also as a critical prerequisite for transforming condition monitoring technologies into viable and sustainable service businesses.

According to the participants, CMS implementation is frequently constrained when service activities remain dispersed across engineering, production, and sales departments without a clear organizational owner. Experts repeatedly highlighted the need for organizational structures capable of leading service development activities, coordinating interactions with customers, building new organizational capabilities, developing business cases, and managing CMS implementation projects over time. Several interviewees also recommended the creation of dedicated service units responsible for CMS development and delivery. These units should possess sufficient autonomy to develop service offerings, coordinate implementation activities, and facilitate collaboration across organizational functions. This means that dedicated service units should be legitimized by OEM management. Legitimized service units provide the authority, resources, and continuity required to develop CMS offerings systematically and align technological capabilities with customer needs and business objectives.

Regarding customer readiness, experts highlighted the importance of demonstrating value through pilot projects and successful implementation examples. Customers become more willing to adopt CMS when they can observe tangible benefits in operational contexts similar to their own. This is connected with solutions proposed regarding CMS value propositions. In this sense, several participants also proposed flexible commercialization mechanisms, including staged investments, subscription models, and financial support arrangements. Such approaches were viewed as effective ways of reducing adoption barriers and lowering the perceived risks associated with CMS investments.

Regarding RQ2, the findings indicate that overcoming CMS implementation challenges requires the simultaneous alignment of value creation mechanisms, service design practices, and service-oriented organizational capabilities. At the value proposition level, experts emphasized the importance of quantifying CMS benefits, developing robust business cases, and tailoring value propositions to multiple stakeholder groups. At the design level, the findings highlight the role of co-creative, iterative, and customer-centred design processes supported by customer segmentation, field observations, and pilot implementations. Finally, at the organizational level, successful implementation requires legitimate service units with multidisciplinary service capabilities, and gradual implementation pathways that reduce customer uncertainty and resistance to change. Taken together, these findings suggest that successful CMS implementation depends on the simultaneous alignment of value creation mechanisms, customer-centred service development processes, and service-oriented organizational capabilities.

### Recommendations for successful CMS implementation

While RQ2 focused on specific solutions for overcoming implementation challenges, the interviews also revealed broader managerial recommendations regarding how OEMs should approach CMS implementation strategically. These recommendations go beyond individual practices and instead reflect guiding principles capable of shaping decision-making, organizational priorities, and resource allocation throughout the development, commercialization, and scaling of CMS offerings.

***Recommendations related to CMS design***

The first recommendation emerging from the interviews is that CMS should be conceptualized and designed as customer-value-driven service offerings rather than technology-driven maintenance solutions. Throughout the interviews, experts consistently emphasized that customers rarely make adoption decisions based on technological sophistication alone. Instead, they evaluate CMS according to their contribution to operational performance, risk reduction, cost efficiency, and broader business objectives. Consequently, OEMs should prioritize understanding how CMS contribute to customer outcomes before determining technological features and functionalities. From this perspective, technological capabilities become enablers of value creation rather than the primary source of value themselves.

A second recommendation concerns the adoption of a co-creative mindset throughout CMS development. The experts repeatedly stressed that successful CMS are not developed internally and subsequently transferred to customers. Rather, they emerge through continuous interactions with customers and other stakeholders. This implies that OEMs should view customers as partners in service development rather than passive recipients of technology. Such partnerships facilitate shared learning, improve the relevance of service offerings, and reduce the risk of developing technologically feasible but commercially unattractive solutions.

A third recommendation is that CMS implementation should be approached as an iterative learning process rather than a predefined linear project. The experts consistently advocated gradual experimentation through pilot projects, prototypes, modular offerings, and phased deployments. Such approaches allow OEMs and customers to learn jointly, validate assumptions, demonstrate value, reduce uncertainty, and progressively adapt service offerings to evolving requirements. Consequently, implementation success depends less on detailed upfront planning than on the organization’s ability to learn and adapt throughout the implementation journey.

***Recommendations related to change management***

The second group of recommendations concerns organizational transformation and change management.

A particularly important recommendation concerns the need to establish organizational legitimacy for service activities within product-centric OEMs. The experts repeatedly described situations where CMS initiatives struggled not because of technical limitations but because service-related activities lacked strategic visibility, authority, and organizational support. Consequently, successful implementation requires OEMs to explicitly recognize CMS as strategic business activities capable of generating customer value and future revenue streams. CMS must therefore be legitimized at the highest organizational levels rather than treated as secondary extensions of product businesses.

Building on this principle, the findings suggest that OEMs should create organizational structures capable of supporting the long-term development and growth of CMS. Dedicated service units emerged as one mechanism for achieving this objective. However, the experts emphasized that the effectiveness of such units depends less on their existence and more on their organizational legitimacy, decision-making authority, and ability to influence activities extending across multiple functions. From this perspective, dedicated service organizations should act as strategic integrators connecting customers, engineering, sales, digitalization, production, and after-sales services.

The findings further indicate that service-oriented capabilities should be viewed as strategic organizational assets rather than operational support functions. Successful CMS implementation requires the integration of technical expertise with competences related to service management, customer engagement, business development, financial analysis, and value communication. Therefore, capability development should be approached as a long-term investment supporting both organizational transformation and sustainable service growth.

Finally, the interviews suggest that CMS implementation should be understood as a dual change-management process involving both providers and customers. Implementation success depends not only on the capabilities of OEMs but also on the readiness of customers to modify existing routines, decision-making processes, and organizational practices. Consequently, OEMs should actively manage organizational change across both organizations by reducing uncertainty, demonstrating value incrementally, and supporting gradual adoption pathways. From this perspective, customer adoption becomes a process of organizational transformation rather than merely a technology purchasing decision.

Overall, the findings related to RQ3 suggest that successful CMS implementation requires a fundamental shift in managerial thinking. Rather than treating CMS as maintenance technologies or digital tools, OEMs should approach them as strategic digital servitization initiatives centred on customer value creation. This requires customer-value-driven service design, legitimate service-oriented organizational structures, multidisciplinary capabilities, and the active management of organizational change across both provider and customer organizations. Consequently, the successful implementation of CMS depends not only on technological capabilities but also on the ability of OEMs to transform how they create, deliver, and capture value through CMS.

## Discussion

### RQ1. What are the main challenges hindering successful CMS implementation?

The findings demonstrate that the principal barriers to successful CMS implementation are not primarily technological but socio-technical in nature. While the CMS literature has traditionally focused on advancing sensing technologies, diagnostics, prognostics, predictive analytics, and maintenance optimization ( Ahmad & Kamaruddin, 2012; Jardine et al., 2006; Selcuk, 2017), the experts consistently identified challenges related to CMS value proposition, service design capabilities, and organizational readiness. These findings suggest that the critical bottleneck in CMS implementation has shifted from technology development towards the organizational capabilities required to successfully implement CMS offerings.

This finding complements and extends previous CMS studies that have acknowledged non-technical barriers, such as organizational resistance, integration challenges, and knowledge-management difficulties ( Ingemarsdotter et al., 2021; van de Kerkhof et al., 2016). Whereas previous studies typically treat these barriers as isolated implementation problems, our findings indicate that they are interconnected and emerge from broader socio-technical misalignments affecting value creation, service development, and organizational transformation. In this sense, the findings move beyond the dominant technical perspective in CMS research and provide a more holistic explanation for implementation failures.

This interpretation aligns with the socio-technical perspective proposed by Münch et al. (2022), who argue that digital servitization success depends on the coordinated development of technological, organizational, and human capabilities. Similar arguments have recently been advanced within Industry 4.0 and digital servitization research, where implementation outcomes have been shown to depend on the balance between technological and organizational factors rather than on technology adoption alone ( Marcon et al., 2022, 2024, 2025). The challenges identified in this study reflect deficiencies across all three capability domains. Consequently, the findings support the view that CMS implementation should be understood as the development of a socio-technical organizational capability system that integrates technological resources, service capabilities, organizational structures, and customer relationships rather than as a technology deployment project.

Importantly, the findings reveal that implementation success is shaped not by technological capabilities alone, but by the alignment of technological, organizational, and relational factors. This contribution advances the CMS literature by demonstrating how value creation, service development, organizational structures, and customer relationships collectively influence implementation outcomes. The findings also provide empirical evidence of how the service paradox materializes in the specific context of CMS implementation. Similar to the machine tool manufacturers studied by Kamp et al. (2023), many experts reported situations in which OEMs invested significantly in monitoring technologies, digital infrastructures, and data-processing capabilities but struggled to achieve customer adoption and profitable revenue streams. These findings reinforce the growing body of literature suggesting that investments in digital technologies do not automatically translate into business value ( Karatzas et al., 2026; Agostini et al., 2026). They also support Rietdijk’s (2026) argument that digital servitization barriers frequently reinforce one another, creating negative synergistic effects that undermine value creation and value capture. In the CMS context, weaknesses in value communication, organizational alignment, customer engagement, and service design appear to interact and amplify implementation difficulties. These findings are particularly relevant in CMS contexts because the value generated by condition monitoring technologies depends on continuous access to operational data, integration with customer maintenance processes, and the ability to demonstrate measurable operational and financial outcomes. Such characteristics make CMS especially sensitive to failures in customer collaboration, value communication, and organizational alignment.

Furthermore, the findings illustrate several of the paradoxical tensions that characterize digital servitization. Consistent with Kohtamäki et al. (2020), experts repeatedly described tensions between product and service orientations, technological sophistication and customer value, and standardization and customization. Similar tensions have recently been identified by Paiola and Grandinetti (2026) and Smania et al. (2026), who argue that paradoxical demands emerge across multiple organizational levels during digital servitization journeys. Our findings suggest that these tensions are not peripheral challenges but structural characteristics of CMS implementation. Rather than disappearing as organizations gain experience, they require continuous balancing and managerial attention.

Therefore, the answer to RQ1 is that the main challenges hindering successful CMS implementation are not technological limitations but multidimensional socio-technical barriers involving value creation, service development, organizational transformation, and customer alignment. Successful implementation depends less on the sophistication of condition monitoring technologies and more on the ability of OEMs to align these interdependent dimensions.

### RQ2. How can OEMs overcome the challenges associated with CMS implementation?

The findings indicate that overcoming CMS implementation challenges requires interventions primarily targeting organizational, relational, and managerial capabilities rather than additional technological investments. This observation reinforces the argument that the successful implementation of CMS depends less on the sophistication of condition monitoring technologies and more on the ability of OEMs to align technologies with value creation mechanisms, service development processes, and organizational capabilities. In this respect, the identified solutions represent practical mechanisms through which firms can address the socio-technical challenges discussed in RQ1.

A first set of solutions concerns value articulation and value quantification. Experts repeatedly emphasized the importance of demonstrating financial and operational benefits through business cases, ROI calculations, performance indicators, and outcome-based narratives. This observation is consistent with prior digital servitization research showing that value demonstration, value appropriation, and value capture remain among the most significant challenges associated with smart service offerings ( Visnjic et al., 2017; Kamp et al., 2023). However, our findings extend this literature by suggesting that value communication should not be viewed as a downstream sales activity but as a core implementation capability. In CMS contexts, customer adoption depends not only on technological performance but also on the ability of OEMs to quantify, communicate, and continuously demonstrate service value. This finding is particularly relevant because CMS generate benefits that are often indirect, future-oriented, and difficult for customers to evaluate prior to adoption.

A second group of solutions focuses on CMS design and development. Experts advocated pilot projects, iterative implementation approaches, customer co-creation activities, and continuous customer involvement throughout the service lifecycle. These findings are consistent with research emphasizing experimentation, service innovation, and agile development practices in digital servitization contexts ( Sjödin et al., 2020; Iriarte et al., 2023). At the same time, they challenge the implicit technology-centred logic found in much of the CMS literature, which frequently conceptualizes implementation as a sequence progressing from data acquisition to diagnostics, prognostics, and maintenance optimization ( Jardine et al., 2006; Ahmad & Kamaruddin, 2012; Selcuk, 2017). Instead, our findings suggest that CMS implementation is better understood as a process of mutual learning in which both OEMs and customers continuously adapt technologies, processes, and expectations. This observation is particularly important in CMS contexts, where value creation depends on continuous access to operational data, integration with customer maintenance processes, and the joint interpretation of equipment performance information. Consequently, customer involvement becomes a prerequisite for implementation rather than simply a desirable design practice.

A third set of solutions concerns service-oriented organizational capability development. Experts stressed the importance of service units with multidisciplinary teams, customer engagement capabilities, financial competencies, and service management expertise. These findings strongly reinforce the capability-based view of digital servitization proposed by Münch et al. (2022) and are consistent with recent evidence indicating that organizational capabilities represent a critical determinant of digital servitization success ( Karatzas et al., 2026; Minaya et al., 2026; Momenzadeh et al., 2026). Importantly, the experts frequently identified capability development as a higher priority than further technological investments. This suggests that many OEMs may currently suffer from organizational capability deficits rather than technology deficits. While condition monitoring technologies have reached relatively high levels of maturity, organizations often lack the structures, competences, and governance mechanisms required to transform technological capabilities into scalable service businesses. Consequently, capability development emerges not only as an implementation solution but also as a foundational condition for CMS implementation.

Interestingly, many of the identified solutions can also be interpreted as coping mechanisms for managing the paradoxical tensions associated with CMS implementation. Pilot implementations, modular service architectures, dedicated service structures, and phased deployment approaches help firms balance competing demands for standardization and customization, flexibility and efficiency, and product and service orientation. As CMS combine technological innovation, service provision, and customer collaboration, these tensions are particularly visible in CMS contexts. This finding directly supports the paradox perspective developed by Kohtamäki et al. (2020) and subsequently extended by Paiola and Grandinetti (2026) and Smania et al. (2026). Rather than eliminating tensions, successful CMS implementation appears to depend on developing organizational practices capable of navigating and balancing them over time.

Therefore, the answer to RQ2 is that successful CMS implementation requires the development of a socio-technical capability system through which technological resources, organizational structures, human competencies, and customer relationships are continuously aligned to support value creation, delivery, and capture. More specifically, OEMs can overcome implementation challenges by strengthening value articulation mechanisms, adopting co-creative and iterative service development practices, and developing service-oriented organizational capabilities. Rather than representing isolated solutions, these practices operate as complementary mechanisms that collectively enable the successful implementation and commercialization of CMS. This finding extends the digital servitization literature by translating broad servitization principles into concrete implementation approaches tailored to the specific realities of CMS.

### RQ3. What recommendations support successful CMS implementation?

The findings suggest that successful CMS implementation should be approached as a digital servitization transformation rather than as the deployment of a condition monitoring technology. While the solutions discussed in RQ2 address specific implementation barriers, the recommendations identified by the experts reveal broader managerial principles capable of guiding the development, commercialization, and scaling of CMS offerings. Collectively, these recommendations indicate that implementation success depends on how OEMs think about CMS, organize around CMS, and manage organizational change rather than on the technological sophistication of the monitoring system itself.

A first managerial recommendation concerns designing CMS around customer value rather than technological functionality. Consistent with digital servitization research ( Baines et al., 2017; Kowalkowski et al., 2017), the findings indicate that sustainable competitive advantage through CMS increasingly derives from delivering customer outcomes rather than selling technological features. Our findings extend this argument by showing that many OEMs continue to approach CMS through engineering-dominated logics, limiting customer acceptance and reducing opportunities for value capture. Successful implementation therefore requires shifting managerial attention from technological performance towards operational improvements, risk reduction, financial outcomes, and customer business objectives. In this sense, technologies should be viewed as enablers of customer value rather than as the primary source of value themselves.

The findings further highlight the importance of adopting a co-creative and iterative service-development logic. Echoing research on agile digital servitization and service innovation ( Sjödin et al., 2020; Iriarte et al., 2023), the experts consistently argued that successful CMS offerings emerge through continuous interactions between providers and customers. However, our findings suggest that co-creation should not be viewed merely as a design tool. Rather, it represents a broader managerial mindset in which customers become active partners in value creation, learning, and service evolution. Similarly, iterative implementation approaches based on pilot projects, modular services, and progressive deployment allow uncertainties to be managed through experimentation and learning. Together, these findings suggest that OEMs should approach CMS implementation as a continuous learning process rather than as a predefined technology project.

A third recommendation concerns the establishment of organizational legitimacy for service activities. Experts consistently argued that CMS implementation requires organizational structures with sufficient authority, visibility, and resources to coordinate activities across engineering, sales, customer support, digitalization, and service functions. This finding extends existing digital servitization research by highlighting legitimacy as a critical but underexplored condition for service growth. Recent work by Kristiansen and Aas (2026) similarly identifies legitimacy as a key enabling mechanism facilitating resource allocation, managerial commitment, and cross-functional collaboration during digital servitization initiatives. Importantly, our findings suggest that legitimacy should not be viewed solely as an outcome of servitization efforts. Rather, it appears to be a prerequisite for successful CMS implementation because organizations must first recognize CMS as strategic service businesses before they can effectively invest in the organizational structures required to support them. Therefore, the findings suggest that legitimized service units help secure resources, managerial support, and cross-functional collaboration, creating the conditions necessary for service development and growth.

Building on this principle, the findings suggest that dedicated service units can play an important role in institutionalizing service-oriented thinking within OEMs. In line with Biesinger et al. (2026), these units should not simply manage service delivery but should function as mechanisms for organizational learning, capability development, and service-business growth. Their role extends beyond coordinating implementation projects and includes connecting customers with internal organizational functions, ensuring the integration of customer knowledge into service development, and supporting the long-term evolution of CMS offerings.

The findings also emphasize the importance of viewing service-oriented capabilities as strategic assets. Echoing Münch et al. (2022), experts highlighted the need to complement engineering expertise with competencies in service design, value-based selling, financial analysis, customer relationship management, and business development. This observation aligns with recent literature showing that digital servitization outcomes depend on the integration of technological and managerial capabilities rather than on technological assets alone ( Agostini et al., 2026; Joshi et al., 2026). Consequently, CMS implementation should be understood as a continuous capability-development process through which firms progressively build the competences required to create, deliver, and capture value from digital services.

Finally, the findings indicate that CMS implementation should be understood as a joint provider-customer transformation process. Consistent with studies emphasizing co-creation, ecosystem collaboration, and relational value creation ( Sjödin et al., 2020; Nguyen et al., 2022a, 2022b; Iriarte et al., 2023), successful implementation requires organizational adaptation on both sides of the relationship. However, our findings extend this literature by demonstrating that customer transformation is not simply a consequence of adoption. The realization of CMS value depends on customers’ willingness and ability to modify routines, maintenance practices, infrastructures, and decision-making processes. As a result, customer adoption should be viewed as an organizational change process rather than a purchasing decision.

Therefore, the answer to RQ3 is that successful CMS implementation requires OEMs to adopt a customer-value-driven service logic, embrace co-creative and iterative service development, establish legitimate service-oriented organizational structures, invest in multidisciplinary service capabilities, and actively manage organizational transformation across both provider and customer organizations. Collectively, these recommendations suggest that CMS implementation should be understood as a digital servitization journey rather than a condition monitoring technology deployment project.

## Conclusions, limitations and future research

### Contributions to theory and practice

This study explored the implementation of CMS by OEMs through an exploratory expert elicitation approach. Specifically, it investigated the challenges limiting CMS implementation, the solutions to overcome these challenges, and the recommendations that can support successful implementation.

Regarding RQ1, the findings demonstrate that the principal barriers to successful CMS implementation are socio-technical. The study identified three interconnected groups of challenges: challenges related to CMS value propositions, challenges associated with CMS design processes, and challenges concerning organizational and customer readiness. Therefore, the findings suggest that the success of CMS is largely determined by an OEM’s ability to create, communicate, and capture value rather than by the maturity of the underlying CM technologies.

Regarding RQ2, the study identified a range of complementary solutions for overcoming these challenges. The findings suggest that successful implementation depends on the simultaneous alignment of value creation mechanisms, co-creative service design processes, and new organizational capabilities. In particular, experts emphasized the importance of quantifying value through business cases and performance indicators, involving customers throughout service development, adopting iterative implementation approaches, developing multidisciplinary service capabilities, and establishing dedicated organizational service-oriented structures responsible for CMS development and delivery. While the digital servitization literature frequently advocates broad approaches such as customer co-creation, organizational capability development, and business model innovation, the present study translates these generic principles into more concrete solutions tailored to the specific implementation realities of CMS by OEMs.

Regarding RQ3, the findings resulted in a set of managerial recommendations that elevate CMS from a maintenance technology initiative to a digital servitization transformation. The experts emphasized the need for OEMs to design CMS around customer value rather than technological functionality, establish dedicated and legitimate service units with sufficient authority and autonomy, develop multidisciplinary capabilities, and actively manage organizational change on both provider and customer sides. Importantly, these recommendations go beyond generic guidance available in the digital servitization literature by addressing issues that are particularly salient in CMS contexts. Consequently, the study provides actionable recommendations specifically targeted at OEMs seeking to develop, commercialize, and scale CMS.

This study contributes to the CMS literature by extending the understanding of CMS implementation beyond technological considerations and highlighting its fundamentally socio-technical nature. The findings support recent digital servitization research arguing that value realization depends on the alignment of technological, organizational, and human capabilities. Furthermore, the study provides empirical evidence of how digital servitization tensions and paradoxes materialize in the specific context of CMS implementation and how OEMs can actively manage them. Importantly, the study complements the broader digital servitization literature, which often examines digitally enabled services at a relatively high level of abstraction, by translating these broader concepts into CMS-specific implementation challenges, solutions, and managerial recommendations. In doing so, the study provides a more contextualized understanding of how digital servitization unfolds in advanced maintenance service environments.

From a managerial perspective, the study offers actionable insights for OEM executives, service managers, innovation managers, and other practitioners involved in the implementation of CMS offerings. The findings suggest that many common implementation pitfalls originate from treating CMS primarily as technological solutions. Such an approach often results in poorly articulated value propositions, insufficient customer involvement, fragmented organizational ownership, weak service capabilities, and difficulties in demonstrating and capturing value. By identifying these pitfalls and proposing corresponding solutions, the study provides practical guidance that can support more effective service development and implementation efforts.

More specifically, the findings encourage OEM practitioners to move beyond a technology-centred implementation logic and adopt a service-oriented perspective focused on customer outcomes and value creation. This represents an important shift from current practices observed by the experts, where CMS initiatives are frequently led by engineering or digitalization functions with limited involvement of service management and customer-facing activities. The proposed recommendations emphasize the creation of legitimate and dedicated service units, the development of multidisciplinary service capabilities, the use of co-creative service design processes, and the active management of organizational change. Such practices can help organizations improve customer adoption, strengthen internal coordination, increase value capture, and reduce the risk of falling into the service paradox frequently reported in servitization research.

Ultimately, the study contributes practical guidance for OEMs seeking to transform condition monitoring technologies into sustainable service businesses. By treating CMS as digital servitization initiatives rather than standalone technology projects, OEMs can improve the effectiveness of implementation efforts, generate greater value for customers, and increase the likelihood of achieving long-term commercial success. More broadly, the findings suggest that the future success of CMS will depend less on further advances in monitoring technologies and more on the ability of OEMs to orchestrate technological, organizational, and relational capabilities into sustainable service-oriented business models.

### Limitations and future research

The findings should be interpreted considering several limitations.

First, the study relies on the perspectives of thirteen experts and therefore reflects their informed perceptions and experiences regarding CMS implementation. Although expert elicitation is particularly suitable for exploring emerging and complex phenomena where knowledge is dispersed among experienced practitioners and researchers, the findings do not allow statistical generalization. Rather than seeking representativeness, the study aims to generate analytically generalizable insights that help explain how experts understand and address CMS implementation challenges. Consequently, the findings should be interpreted as informed expert knowledge rather than as evidence of the prevalence of specific challenges or practices across the broader population of OEMs.

Second, the study focuses specifically on CMS implementation by OEMs. While several findings may be relevant to other digitally enabled services, caution should be exercised when extending the conclusions to different forms of digital servitization. CMS possess distinctive characteristics. These characteristics may generate implementation challenges and organizational requirements that differ from those associated with other digital services. Therefore, the transferability of the findings to other digital servitization contexts should be assessed carefully and validated through additional research.

Third, regarding triangulation and participant validation, we acknowledge that the study did not employ multiple data sources or formal participant validation procedures. The findings are based exclusively on expert interviews and therefore capture how experts interpret, explain, and make sense of CMS implementation challenges. Consequently, some findings may reflect retrospective interpretations, individual experiences, or context-dependent viewpoints that could differ from observations obtained through direct organizational studies.

A further limitation is the cross-sectional nature of the study. The interviews provide a snapshot of expert experiences and reflections at a particular point in time but do not capture how implementation processes develop over extended periods. Given that CMS implementation often involve long-term organizational transformation, future longitudinal studies could provide valuable insights into how capabilities, organizational structures, customer relationships, and implementation outcomes evolve throughout different stages of the servitization journey.

Taken together, these limitations suggest that future research should complement expert-based insights with longitudinal, multi-stakeholder, and case-based investigations capable of examining CMS implementation processes as they unfold in practice.

Several opportunities for future research emerge from this study.

First, future studies could examine CMS implementation longitudinally to better understand how implementation challenges, organizational capabilities, and customer relationships evolve over time and across different stages of digital servitization. Such studies would help explain not only what challenges occur, but also how organizations successfully overcome them and how socio-technical capabilities develop throughout the implementation journey.

Second, additional research could investigate the role of dedicated service units in greater depth. In particular, future studies could explore how organizational legitimacy for service activities is established, maintained, and strengthened within product-centric manufacturing organizations. Such work could provide a deeper understanding of the organizational mechanisms that support the implementation of CMS.

Third, future studies could compare different sectors, firm sizes, and digital service types to identify the contextual conditions under which particular implementation practices are more or less effective. Such comparative analyses would contribute to refining managerial recommendations and developing more context-sensitive implementation frameworks.

Fourth, future research could investigate the interaction between value creation, value communication, and value capture in CMS contexts. Given the importance of value quantification identified in this study, further research could explore how OEMs develop business cases, pricing models, and commercialization strategies that support customer adoption while ensuring sustainable value capture.

Finally, future studies could expand the perspective beyond OEMs by incorporating customers, technology providers, and ecosystem partners. Multi-stakeholder studies could provide a richer understanding of how collaboration, trust, data governance, and organizational change influence the implementation and scaling of CMS across organizational boundaries.

## Data Availability

All research data underlying the results of this article—including the call for participants, consent forms, interview guides, and fully manually anonymised interview transcripts—are available on Mendeley Data under the terms of the Creative Commons Attribution 4.0 International License (CC BY 4.0). The interviews were transcribed using Tactiq; no other software was used for data processing or analysis. Mendeley: Overcoming Challenges in Implementing Condition Monitoring Services: Recommendations from an Expert Elicitation Exploratory Study, 10.17632/tf74rr4jsj.1 ( Iriarte et al., 2026). This project contains the following extended data:
• call for participants, consent forms, interview guides, interview transcripts, and data analysis. Data is available under the terms of the Creative Commons Attribution 4.0 International License (CC BY 4.0).
