# Supplementary material for: Overcoming Challenges in Implementing Condition Monitoring Services: Recommendations from an Expert Elicitation Exploratory Study
Source: Open Res Eur. 2026 Aug 4;5:151. Originally published 2025 May 29. [Version 2] doi: 10.12688/openreseurope.20044.2 (PMC13560564; doi:10.12688/openreseurope.20044.2)
Supplement: Peer review history

# Reviewer report: Reviewer response for version 2

**DOI:** 10.21956/openreseurope.26701.r78566

**Published:** 2026-08-12

**Authors:** Muztoba Ahmad Khan

**Affiliations:** 1 Carroll University, Waukesha, Wisconsin, USA

The authors have addressed my main concerns. The theoretical background is now much better aligned with the findings. The methodology is also significantly clearer now that the authors have provided needed detail on expert selection, the coding process, and thematic saturation. I am satisfied with the revisions and have no further major comments.

Is the study design appropriate and does the work have academic merit?

Partly

Is the work clearly and accurately presented and does it cite the current literature?

Yes

If applicable, is the statistical analysis and its interpretation appropriate?

Not applicable

Are all the source data underlying the results available to ensure full reproducibility?

Partly

Are the conclusions drawn adequately supported by the results?

Yes

Are sufficient details of methods and analysis provided to allow replication by others?

Partly

Reviewer Expertise:

My area of expertise is servitization.

I confirm that I have read this submission and believe that I have an appropriate level of expertise to confirm that it is of an acceptable scientific standard.

# Reviewer report: Reviewer response for version 2

**DOI:** 10.21956/openreseurope.26701.r78567

**Published:** 2026-08-12

**Authors:** Shaun West

**Affiliations:** 1 Lucerne University of Applied Sciences and Arts, Horw, Switzerland

Overall assessment::

 Version 2 represents an improvement over the original manuscript. The authors have responded to the earlier reviews and have revised the theoretical background, methodology, findings, discussion and conclusions.

 The manuscript now adopts a socio-technical perspective and positions CMS implementation within digital servitization.

 It provides a clearer definition of CMS implementation, explains the relationship between CMS and PSS, identifies OEMs as the main target group, and presents a more coherent research gap. The methodology is also more transparent: the authors explain the expert selection criteria, expand the participant profiles, describe the coding process, and provide an explicit saturation assessment. The findings and discussion are now organised around the three research questions, while the conclusion presents clearer theoretical and managerial contributions.

 These revisions address most of my original concerns. However, several reservations remain, principally concerning the empirical support presented in the Findings section, the depth with which the theoretical lenses are applied, the composition of the expert sample and the distinction between solutions and recommendations.

 Major reservations

 1. Empirical evidence in the Findings section

 Although the Findings section is now considerably easier to follow, it contains almost no direct evidence from the interviews. The authors have moved the coding tables and illustrative quotations to the Extended Data. This improves readability, but it also removes participants' voices from the main manuscript. Statements such as “experts consistently emphasised” and “interviewees repeatedly noted” are difficult to assess without representative quotations or some indication of how perspectives varied across participants. For a qualitative study, the principal empirical claims should be demonstrably grounded in the data presented within the article.

 The authors should include at least one, and preferably two, appropriately contextualised quotations for each higher-order theme, identified by participant number and background. In particular, quotations from OEM participants should be distinguished from those of university and research-centre participants. Contrasting or dissenting perspectives should also be reported where they occurred.

 2. Application of the theoretical lenses

 The introduction and theoretical background now establish socio-technical systems theory and the perspective of the servitization paradox. This is a considerable improvement. Nevertheless, the Discussion often uses these theories to label or confirm the findings rather than to generate a deeper explanation of them. For example, the manuscript claims to provide empirical evidence of servitization paradoxes. Still, the Findings section does not clearly identify the competing demands, organisational tensions or coping practices reported by participants. Likewise, the three principal categories—value proposition, design process, and organisational and customer readiness—are not systematically mapped onto the technological, organisational and human dimensions of the socio-technical lens.

 The theoretical contribution would be stronger if the authors provided an integrative framework or table showing:

 * how the empirical themes relate to socio-technical capabilities;

 * which paradoxical tensions arise during CMS implementation;

 * how the proposed solutions respond to those tensions; and

 * what is specifically distinctive about CMS compared with digital servitization generally.

 Without this additional synthesis, the contribution remains valuable but primarily descriptive and confirmatory.

 3. Sampling and transferability

 The expert-selection process and participant profiles are much clearer. However, the study focuses on CMS implementation by OEMs, whereas only four of the 13 participants are directly employed by OEMs. The remaining participants offer relevant expertise, but their experience is primarily academic, consultative or based on collaborative research projects. The authors should therefore moderate claims that the findings establish the “principal barriers” experienced by OEMs. They should more precisely represent a synthesis of expert perceptions about those barriers. If confidentiality permits, the authors should also report the number of distinct OEMs represented and provide aggregate information about industrial sectors, company sizes and participants’ direct involvement in completed CMS implementations. The concept of analytical generalisation is now introduced, but the limits of transferring findings from this heterogeneous expert sample to OEM

 practice should remain explicit throughout the discussion and conclusion.

 4. Saturation and analytical transparency

 Table 3 is a welcome improvement and more defensible than assuming saturation based on the number of interviews. Nevertheless, presenting interviews in three aggregated rounds obscures the evolution of the analysis. The authors should clarify whether the expert numbering reflects the chronological order of interviews, how the decision to stop data collection was made, and whether any additional potential participants remained available. An interview-by-interview cumulative grid would provide stronger evidence than the current three-round summary. The column labelled “new statements” also appears to count coded excerpts rather than genuinely novel concepts. It should be renamed or explained. The authors should distinguish between code saturation and meaning saturation, particularly because the final three interviews still generated 27 coded statements. Finally, the manuscript describes the method as reflexive thematic analysis while also emphasising independent coding and consensus. The authors should clarify their analytical position and briefly address researcher reflexivity and the ways in which the researchers’ backgrounds influenced coding and interpretation.

 5. Distinction between RQ2 and RQ3

 The distinction between “solutions” under RQ2 and “recommendations” under RQ3 remains less clear than the manuscript suggests. Pilot projects, customer co-creation, multidisciplinary capabilities, service units and change management appear in both sections. A challenge–solution–recommendation mapping would help demonstrate the analytical relationship between these categories and reduce repetition. The authors should explain whether recommendations represent a higher level of abstraction, a prioritised set of actions or a separate class of empirical findings.

 Minor revisions

 The manuscript is much clearer than Version 1, but another careful language edit is required. Examples include:

 * The title would read more naturally as Overcoming Challenges in Implementing Condition Monitoring Services: An Exploratory Expert-Elicitation Study and Recommendations for OEMs.

 * On p. 7, “Tagscherer and Carbon (2023) highlights” should be “highlight”.

 * On p. 8, remove the duplicated full stop after “existing literature”.

 * On p. 8, RQ3 should be rewritten as: “What recommendations should OEMs follow to implement CMS successfully?”

 * On p. 10, revise expressions such as “research centres experts” and “OEMs participants”.

 * On p. 22, “CMS implementation often involve” should be “CMS implementation often involves”.

 * The wording of the research questions should remain identical in the theoretical background, findings and discussion.

 Summary

 Version 2 is a stronger manuscript and now has a recognizable theoretical foundation, a clearer methodological account and a more coherent contribution. Most of my original concerns have been addressed. The remaining reservations concern how convincingly the empirical evidence supports the analysis and how fully the theoretical lenses are used to explain the findings.

 I therefore recommend Approved with reservations, subject to strengthening the presentation of interview evidence, moderating claims based on the sample, clarifying the saturation assessment, and sharpening the theoretical synthesis.

Is the study design appropriate and does the work have academic merit?

No

Is the work clearly and accurately presented and does it cite the current literature?

Partly

If applicable, is the statistical analysis and its interpretation appropriate?

Not applicable

Are all the source data underlying the results available to ensure full reproducibility?

Partly

Are the conclusions drawn adequately supported by the results?

No

Are sufficient details of methods and analysis provided to allow replication by others?

Partly

Reviewer Expertise:

NA

I confirm that I have read this submission and believe that I have an appropriate level of expertise to confirm that it is of an acceptable scientific standard, however I have significant reservations, as outlined above.

# Reviewer report: Reviewer response for version 1

**DOI:** 10.21956/openreseurope.21680.r64263

**Published:** 2025-12-29

**Authors:** Muztoba Ahmad Khan

**Affiliations:** 1 Carroll University, Waukesha, Wisconsin, USA

This manuscript addresses the challenges of OEMs in provisioning condition monitoring services. This is an important topic in digital servitization and this study provides useful practitioner insights. However, the following issues limit the strength of the work and should be addressed.

 The authors mention that CMS challenges “go beyond technical aspects,” yet the theoretical background section is dominated by technical descriptions (condition monitoring phases, failure modes, types of analytics). The organizational change and business model literature are included superficially. This creates a mismatch between the theory section (which is descriptive and technology heavy) and the findings (which emphasize business model issues, value propositions, and organizational readiness).

 I feel that the sampling strategy is not described in enough detail. It is unclear how experts were identified and why the final group reflects the intended population. Table 1 shows a heavy dominance of researchers and research-center personnel, raising questions about whether the insights truly represent OEM challenges in a real-world sense. Only four OEM experts appear in the sample. This undermines the generalizability of the findings, especially given that CMS implementation difficulties are organizational and context-dependent, not merely conceptual.

 The manuscript repeats Guest et al.’s (2006) finding that twelve interviews often reach saturation but does not demonstrate that saturation actually occurred in this study. The authors didn’t provide saturation grid or coding evolution. Instead, the authors assume saturation because they conducted 13 interviews. This is not methodologically defensible.

 I also found the coding process difficult to evaluate because the paper does not show how codes were generated or refined. More transparency is needed to support the validity of the findings.

Is the study design appropriate and does the work have academic merit?

Partly

Is the work clearly and accurately presented and does it cite the current literature?

Yes

If applicable, is the statistical analysis and its interpretation appropriate?

Not applicable

Are all the source data underlying the results available to ensure full reproducibility?

Partly

Are the conclusions drawn adequately supported by the results?

Yes

Are sufficient details of methods and analysis provided to allow replication by others?

Partly

Reviewer Expertise:

My area of expertise is servitization.

I confirm that I have read this submission and believe that I have an appropriate level of expertise to confirm that it is of an acceptable scientific standard, however I have significant reservations, as outlined above.

# Response

**Published:** 2026-08-04

**Authors:** Ion Iriarte

**Affiliations:** Design Innovation Center, Mondragon University Higher Polytechnic School, Arrasate, Basque Country, Spain

We thank the reviewer for these valuable observations. We agree that the previous version of the manuscript would benefit from a stronger alignment between the theoretical framing, the empirical findings, and the methodological description. In response, we have substantially revised the manuscript.

**Theoretical background** We comprehensively revised and restructured the theoretical background to better align with both the research objectives and the empirical findings. First, we significantly reduced the technical description of CMS and retained only the material necessary to define CMS and position it within the context of digital servitization and Product-Service Systems (PSS). This allowed us to devote considerably more attention to the organizational, managerial, and business dimensions of implementation. Second, we expanded the discussion of CMS from a digital servitization perspective. The revised manuscript now conceptualizes CMS as digitally enabled service offerings implemented by OEMs to support customer value creation and servitization strategies. This strengthens the link between CMS, PSS, digital servitization, and the business-oriented findings of the study. Third, we introduced a socio-technical systems perspective (Münch et al., 2022) as the primary theoretical lens. This perspective allows CMS implementation to be interpreted as a process requiring the alignment of technological, organizational, and human capabilities rather than as a purely technological challenge. This theoretical lens also enabled us to interpret the identified challenges, solutions, and recommendations as manifestations of broader socio-technical capability development processes rather than isolated implementation problems. Fourth, we developed two new subsections entitled “Challenges in CMS implementation” and “Approaches to successful CMS implementation.” These sections substantially strengthen the discussion of organizational transformation, capability development, customer collaboration, business model challenges, value capture, leadership, organizational legitimacy, and servitization tensions. In particular, the revised manuscript now analyses the service paradox as a manifestation of competing demands associated with customer value creation, service monetization, resource allocation, and organizational transformation. As a result, the revised theoretical background now more closely reflects the socio-technical and digital servitization challenges identified by the experts and provides a clearer foundation for discussing value propositions, organizational readiness, customer collaboration, service development, and business model innovation.

**Methodology** We substantially revised the methodological section to improve transparency regarding expert selection, coding procedures, and saturation assessment. Regarding the sampling strategy, we expanded the expert selection subsection to clearly explain: how experts were identified; the inclusion criteria used; the rationale for purposive sampling; the expertise required for participation; the relevance of each participant to the research objectives. We further clarify that the objective of the study was not to collect perspectives exclusively from OEM employees but to capture expert knowledge regarding CMS implementation from multiple complementary viewpoints. CMS implementation is inherently multidisciplinary, involving technological, organizational, service, and business-model dimensions. We acknowledge that only four participants were employed directly by OEMs. However, the remaining experts were intentionally selected because of their extensive involvement in OEM-focused servitization, CMS, and Industry 4.0. In particular, the research-centre experts have accumulated experience across multiple OEM implementation contexts through long-term knowledge-transfer and industrial collaboration projects. Their perspectives therefore provide insights derived from numerous implementation experiences rather than from a single company. To make this clearer, we substantially expanded the expert descriptions in Table 1 and now explicitly acknowledge this characteristic of the sample in the limitations section. Regarding the coding process, we substantially improved transparency. The revised data analysis section now explains the progression from: raw interview data, meaningful statements, preliminary categories, first-order themes, and second-order themes. We also describe how two researchers independently examined the material and iteratively refined codes and themes through analytical discussions and consensus-building. In addition, the complete coding structure, including first-order themes, second-order themes, and illustrative quotations, is now provided in the Extended Data.

 This improves traceability between the empirical material and the findings reported in the manuscript. Regarding thematic saturation, we agreed that the previous version did not provide sufficient evidence. The revised manuscript therefore no longer assumes saturation based solely on the number of interviews conducted. Instead, we introduced a dedicated subsection entitled “Data Saturation” and added Table 3, which documents the emergence of new meaningful statements, first-order themes, and second-order themes across successive analytical rounds. The analysis shows that no new first-order or second-order themes emerged during the final round of interviews, providing empirical evidence that thematic saturation had been achieved.

 We believe these revisions have significantly strengthened the manuscript by improving the alignment between the theoretical framing, methodological approach, and empirical findings. Together, these changes enhance the rigor, coherence, credibility, and explanatory power of the study while providing a clearer contribution to both CMS and digital servitization research.

 We are grateful for the reviewer’s comments, which have significantly contributed to improving the clarity, theoretical grounding, and methodological robustness of the manuscript.

# Reviewer report: Reviewer response for version 1

**DOI:** 10.21956/openreseurope.21680.r64266

**Published:** 2025-11-22

**Authors:** Shaun West

**Affiliations:** 1 Lucerne University of Applied Sciences and Arts, Horw, Switzerland

**Paper Title:** *Overcoming challenges in implementing condition monitoring services: expert elicitation*

**Authors:** Ion Iriarte, Hien Nguyen Ngoc, Ganix Lasa

**Journal:** Open Research Europe

**Version:** 1

**Review Decision:** Reject

 1. Title

 The title clearly states the topic and method, referencing condition monitoring services and expert elicitation. It is specific, relevant, and academically appropriate.

**Areas to Improve:**

- Add clarity about the industry or context (e.g., OEMs or industrial services).
- Consider explicitly labelling this as a qualitative or exploratory study.
- Reframe using more active voice to create a sharper impression.
- The phrasing is slightly abstract — stronger verbs would help.
- If the paper proposes solutions, signal this in the title.

2. Abstract

 The abstract provides a general overview of the topic, stating that implementation challenges are primarily organisational. However, it lacks specificity and does not preview key findings or contributions.

**Areas to Improve:**

- Please include the number of participants and their profiles.
- Present at least one concrete finding or insight.
- Clearly state the research questions or objective.
- Replace vague phrases like “draws on insights” with specific outcomes.
- Use a stronger, more conclusive final sentence.

3. Introduction

 The introduction references IoT and servitization, noting the struggle to implement CMS despite being technically ready. Three research questions are listed. However, the structure and clarity are weak.

**Areas to Improve:**

- The opening sentence is cluttered and poorly written, lacking clarity.
- Key terms, such as “condition monitoring services,” are not clearly defined.
- The narrative lacks cohesion between background, problem, and purpose.
- There is little effort to motivate the research with practical or theoretical urgency.
- Prior work is mentioned but not critically reviewed or contrasted.

4. Literature Review / Theoretical Background

 The section outlines layers of CMS and technical/organisational barriers. It includes relevant literature but is overly descriptive and lacks analytical depth.

**Areas to Improve:**

- Fails to define a focused research gap until the end.
- Key theoretical foundations are not well-articulated.
- Lacks synthesis or critique of prior studies.
- Organising the literature thematically would help guide the reader effectively.
- The “service paradox” is mentioned but not analysed.

5. Methodology

 Semi-structured interviews were conducted with 13 European experts. Sampling strategy and thematic analysis are mentioned, but without sufficient rigour or detail.

**Areas to Improve:**

- No explanation is provided for why this method is most suitable for addressing the research questions.
- Lacks a process flow or structure for how data was analysed.
- Interview questions or prompts are not included.
- No mention of coding software or reliability strategies.
- Thematic analysis is described vaguely and lacks transparency.

6. Results

 Research questions organise results, and themes are presented in tables with supporting quotes. Findings indicate challenges in organisational integration and customer alignment.

**Areas to Improve:**

- Tables are overly complex and challenging to interpret.
- Quotes are short and lack context or analytical framing.
- The connection between themes and research questions is not always clear.
- No evidence of data triangulation or participant validation is provided.
- Over-reliance on first-order coding categories weakens clarity.

7. Discussion

 The discussion reiterates existing findings and re-engages with the literature, but lacks analytical depth. There is minimal theoretical development or critique.

**Areas to Improve:**

- Does not explicitly answer all research questions.
- No critical engagement with contradictions, limitations, or failures.
- Lacks a comparative discussion with similar studies.
- Does not address how findings reduce or close the identified research gap.
- Claims of contribution are not adequately supported.

8. Conclusions and Limitations

 Recommendations are offered, and the paper concludes that organisational issues are the main challenge. Limitations are acknowledged briefly.

**Areas to Improve:**

- The conclusion does not restate or clearly tie back to the research questions.
- The first paragraph is vague and poorly structured.
- Limitations are acknowledged without critical reflection.
- Future research directions are too general and lack actionable implications.
- The conclusion lacks a clear, final message of value.

English Language and Clarity

**Clarity Issues:**

- The text frequently employs passive voice and overly complicated phrasing.
- The writing violates clarity principles, including long sentences, imprecise words, and a lack of punch.
- The paragraph flow is uneven, particularly in the introduction and discussion sections.
- Several sentences are jargon-filled and would benefit from being rewritten in plain language.
- Overall readability is low and needs extensive editing to meet indexing standards.

Final Decision: Reject

 This paper addresses a relevant problem area but falls short in execution across all core academic criteria: clarity, theoretical foundation, methodological rigour, and structured argumentation. The writing lacks precision, the literature review is insufficiently critical, and the results are poorly interpreted. The research questions are not convincingly answered, and the claimed contributions are vague.

Is the study design appropriate and does the work have academic merit?

No

Is the work clearly and accurately presented and does it cite the current literature?

Partly

If applicable, is the statistical analysis and its interpretation appropriate?

Not applicable

Are all the source data underlying the results available to ensure full reproducibility?

Partly

Are the conclusions drawn adequately supported by the results?

No

Are sufficient details of methods and analysis provided to allow replication by others?

Partly

Reviewer Expertise:

PSS innovation - I have in the past developed and supported the develment of condition based mainteance services.

I confirm that I have read this submission and believe that I have an appropriate level of expertise to state that I do not consider it to be of an acceptable scientific standard, for reasons outlined above.

# Response

**Published:** 2026-08-04

**Authors:** Ion Iriarte

**Affiliations:** Design Innovation Center, Mondragon University Higher Polytechnic School, Arrasate, Basque Country, Spain

**Title:** We thank the reviewer for this suggestion. In response, we have revised the title to better reflect the study's objectives, methodology, and contribution: *“Overcoming Challenges in Implementing Condition Monitoring Services: Recommendations from an Expert Elicitation Exploratory Study”* The revised title explicitly emphasizes: the focus on overcoming implementation challenges; the practical contribution in terms of recommendations; the use of an expert elicitation exploratory approach. We believe that the revised title provides a clearer and more informative description of the study while better reflecting its empirical and exploratory nature.

**Abstract:** We thank the reviewer for these constructive observations. To address these concerns, we completely revised the abstract. The revised abstract now:

- Clearly states the objective of the study, namely, to identify the challenges limiting CMS implementation, the solutions used to overcome them, and the recommendations supporting successful implementation by OEMs.
- Specifies the participant sample, indicating that the study is based on 13 experts from OEMs, universities, research centres, and consultancy organizations with expertise in CMS, digital servitization and Industry 4.0.
- Replaces general wording with a more explicit description of the research approach and findings.
- Presents concrete findings, including the identification of three major groups of implementation challenges well as the corresponding solutions and recommendations.
- Concludes with a stronger statement emphasizing both the theoretical contribution to CMS and digital servitization literature and the practical guidance provided to OEMs seeking to implement CMS.

We believe that the revised abstract now provides a more informative and representative overview of the study and addresses all the concerns raised by the reviewer.

**Introduction:** We have substantially revised the Introduction to improve clarity, coherence, theoretical positioning, and justification of the study. First, the opening section has been completely rewritten to provide a clearer and more logical progression from Industry 4.0 and digital servitization to the growing strategic importance of Condition Monitoring Services (CMS) for OEMs. This restructuring improves readability and establishes a stronger context for the study. Second, we strengthened the definition and explanation of CMS. The revised manuscript now explicitly presents CMS as digitally enabled service offerings through which OEMs create value by combining condition monitoring technologies, data analytics, and service activities to support customer outcomes throughout the equipment lifecycle. Third, we improved the narrative flow by explicitly connecting the emergence of CMS within digital servitization, the implementation difficulties experienced by OEMs, the limitations of the existing literature, and the resulting research gap addressed by this study. This creates a clearer link between the background, the problem statement, and the study objectives.   Fourth, we strengthened the practical and theoretical relevance of the study. The revised introduction now emphasizes that, despite significant technological advances, many CMS initiatives fail to achieve their intended outcomes because implementation challenges extend beyond technical factors and involve organizational, managerial, and relational issues. This provides a stronger justification for investigating implementation challenges from a digital servitization perspective. Finally, we expanded the review of prior research to move beyond a descriptive overview of technical CMS challenges. The revised introduction now contrasts the predominantly technology-oriented CMS literature with emerging socio-technical digital servitization perspectives. This comparison highlights the disconnect between the technical focus of existing CMS studies and the broader organizational and managerial challenges identified in digital servitization research. At the same time, the revised introduction acknowledges that the digital servitization literature typically examines digitally enabled services at a relatively general level of abstraction and therefore provides limited guidance regarding the specific implementation challenges associated with CMS. This distinction helps justify the need for a dedicated investigation of CMS implementation and provides a clearer rationale for the study and its research questions. We believe these revisions have substantially improved the clarity, coherence, theoretical grounding, and motivation of the Introduction while providing a clearer justification for the study’s focus on CMS-specific implementation challenges, solutions, and recommendations

**Theoretical Background** We substantially revised and reorganized the entire theoretical background section. First, we restructured the literature review around thematic sections. The revised theoretical background is now organized around: (i) a socio-technical perspective on CMS implementation within digital servitization, (ii) challenges in CMS implementation, (iii) approaches to successful CMS implementation, and (iv) the research gap. This structure provides a clearer narrative flow and aligns the theoretical background more closely with the study objectives and research questions. Second, we clarified the concept of CMS implementation. The revised theoretical background now explicitly conceptualizes implementation as the organizational process through which OEMs develop, deliver, integrate, and scale CMS-based service offerings within a digital servitization strategy. Third, we strengthened the theoretical foundations of the study by incorporating socio-technical systems theory. In particular, we introduced the work of Münch et al. (2022) to frame CMS implementation as a socio-technical phenomenon requiring the alignment of technological, organizational, and human capabilities. We also incorporated the servitization paradox perspective proposed by Kohtamäki et al. (2020), which helps explain the competing demands that organizations face during servitization and digital transformation. Fourth, we substantially revised and expanded the subsection on CMS implementation challenges. Rather than simply mentioning the service paradox, the revised manuscript now analyses it as a manifestation of competing organizational demands associated with value creation, service provision, customer relationships, and resource allocation. This perspective helps explain why technologically mature CMS initiatives often fail to achieve their intended business outcomes. Fifth, we moved beyond a descriptive review of prior studies and introduced a more critical synthesis of the literature. The revised manuscript contrasts the dominant technology-oriented CMS literature with emerging research in digital servitization emphasizing organizational capabilities, service development, customer collaboration, organizational transformation, and business model innovation. This provides a stronger basis for identifying the limitations of current knowledge and positioning the contribution of the study. Finally, the research gap is now introduced progressively throughout the literature review and culminates in a clearly articulated gap before the presentation of the research questions. Rather than arguing that knowledge is absent, the revised manuscript acknowledges existing contributions while highlighting that current CMS research remains predominantly technology-oriented and that digital servitization research discusses CMS implementation challenges and solutions at a relatively high level of abstraction treating digital services in a relatively homogeneous manner. This positioning provides a stronger justification for examining the specific implementation challenges, solutions, and recommendations associated with CMS. We believe that these revisions substantially strengthen the theoretical foundations, analytical depth, thematic coherence, and overall contribution of the theoretical background section.

**Methodology** We substantially revised the Methodology section to improve transparency, analytical rigor, and reproducibility. First, we expanded the justification for the research approach. In the revised manuscript, we explain that CMS implementation constitutes a complex socio-technical phenomenon involving multiple dimensions. While previous studies have identified individual technical or organizational barriers, knowledge about how these challenges are experienced, interpreted, and managed during real-world CMS implementation remains fragmented. Consequently, an exploratory qualitative design is particularly appropriate because it allows the investigation of a complex phenomenon where contextual factors are critical and where existing theory provides only a partial explanation of implementation practices. We further explain that expert elicitation is especially suitable in such situations because it enables access to experience-based knowledge, tacit understanding, and practical insights held by individuals directly involved in the phenomenon under study. Therefore, expert interviews were selected as the most appropriate method for generating context-rich insights into the challenges, solutions, and implementation practices associated with CMS by OEMs. Second, we completely revised the Data Analysis section and introduced a structured and transparent analytical procedure. The revised manuscript now describes the full progression from interview transcripts to meaningful statements, preliminary categories, first-order themes, and second-order themes. In addition, we provide a dedicated analytical flow table (Table 2) summarizing each stage of the analysis process. Third, we clarified that the interview protocol and guiding questions are available through the Extended Data repository (DOI provided in the manuscript). The revised text explicitly describes how the interview guide was structured around the three research questions and how follow-up questions were used to explore emerging themes. Fourth, we strengthened the discussion of analytical rigor and trustworthiness. The manuscript now explains that all coding was conducted manually by two researchers through an iterative process of independent review, discussion, refinement, and consensus-building. This procedure was adopted to enhance the credibility, consistency, and transparency of the findings. Finally, we substantially expanded the description of the thematic analysis. We introduced a dedicated Data Saturation subsection and Table 3 to demonstrate saturation rather than assuming it based solely on sample size. Table 3 documents the emergence of new meaningful statements, first-order themes, and second-order themes across successive analytical rounds. The results show that although the final interviews provided additional examples and contextual variation, no new first-order or second-order themes emerged during the final analytical round. This pattern indicates that the thematic structure had stabilized, providing empirical evidence that thematic saturation had been achieved. We believe these revisions significantly improve the transparency, methodological rigor of the study and provide readers with a clearer understanding of how the findings were derived from the empirical data.

**Results/Findings** We substantially revised the entire Findings section. First, we simplified the presentation of the findings by moving the detailed coding tables (previously included in the main manuscript) to the Extended Data (Tables E1–E5). This allows interested readers to access the complete coding structure while improving the readability of the manuscript. The revised Findings section now focuses on higher-level themes and their interpretation rather than on detailed coding outputs. Second, we completely restructured the Findings section around the three research questions. The revised section is now organized into: Challenges in CMS implementation (RQ1), Solutions to CMS implementation challenges (RQ2), and Recommendations for successful CMS implementation (RQ3). This revision strengthens the connection between the empirical findings and the study objectives while improving the traceability of the results. Third, we reduced the emphasis on first-order coding categories and instead organized the findings around broader analytical themes. This thematic structure provides a clearer analytical narrative and makes the relationships between challenges and proposed solutions more explicit. Fourth, the recommendation section was substantially revised and elevated into a set of managerial propositions for successful CMS implementation. While the solutions section (RQ2) now focuses on specific implementation practices, while the recommendations section (RQ3) presents broader principles related to CMS design and organizational change management. This revision provides clearer analytical differentiation between solutions and recommendations and strengthens the managerial contribution of the study. Regarding triangulation and participant validation, we acknowledge that the study did not employ multiple data sources or formal participant validation procedures. This choice is consistent with the exploratory expert elicitation approach adopted in the study, whose primary objective was to capture and analyse expert knowledge and experience regarding CMS implementation. To enhance the trustworthiness and credibility of the findings, we instead relied on several established qualitative research practices, including purposive expert selection, a multidisciplinary expert sample, systematic thematic analysis, independent review and discussion of codes by two researchers, the provision of the complete coding structure in the Extended Data, and the assessment of thematic saturation as documented in the new Data Saturation subsection. Collectively, these measures were implemented to strengthen the dependability, transparency, and credibility of the findings. Overall, we believe that the revised Findings section is substantially clearer, more analytically focused, and more directly connected to the research questions, while the complete coding structure remains available through the Extended Data to ensure transparency and traceability. **Discussion** We substantially revised and restructured the Discussion around the three research questions (RQ1–RQ3) and strengthened its connection to both the CMS and digital servitization literatures. Specifically:

- Explicitly answering all research questions. The Discussion has been reorganized into three dedicated subsections corresponding to RQ1, RQ2, and RQ3. Each subsection now concludes with an explicit answer to the respective research question, synthesizing the findings and their implications. This structure improves clarity and ensures that all research questions are directly addressed.
- Stronger theoretical development and analytical depth. The revised Discussion moves beyond describing findings and provides a deeper theoretical interpretation of the findings. This allowed us to explain why CMS implementation challenges occur, how they can be approached, and how this relates to broader theoretical debates within CMS and digital servitization literature.
- Stronger comparison with previous studies. We substantially expanded the comparison of our findings with both CMS and digital servitization research. The revised Discussion contrasts our findings and positions the results alongside recent studies.
- Clarification of how the findings address the research gap. The revised Discussion now explicitly argues that the study addresses a gap in CMS research concerning the organizational, managerial, and service-related dimensions of CMS implementation.
- Stronger and better-supported contributions. The contributions have been clarified and are now supported through the discussion of the findings and current literature.

**Conclusion, Limitations and Future Research** We substantially revised the entire section to strengthen its structure, analytical depth, and contribution. Specifically:

- We restructured the conclusion around the three research questions (RQ1–RQ3) and explicitly restated the main findings associated with each question. The revised conclusion now clearly summarizes the identified implementation challenges, proposed solutions, and managerial recommendations, thereby strengthening the connection between the research objectives, findings, and study outcomes.
- We completely rewrote the opening paragraph of the conclusion to provide a clearer overview of the study purpose, scope, and contribution. The revised opening now situates the study within the broader context of CMS implementation and digital servitization while clearly summarizing the focus of the research.
- We significantly expanded the discussion of theoretical and managerial contributions. Furthermore, the revised conclusion highlights how the study translates broad digital servitization concepts into more concrete implementation solutions and recommendations tailored to the specific characteristics of CMS.
- We strengthened the limitations section by providing a more critical reflection on the implications of the expert elicitation approach, the focus on CMS implementation by OEMs, the absence of multiple data sources and formal participant validation procedures, and the cross-sectional nature of the study. Rather than simply listing limitations, the revised manuscript discusses how these factors influence the interpretation and transferability of the findings.
- We revised the future research section to provide more specific and actionable research directions. The revised manuscript now proposes longitudinal studies examining how CMS implementation evolves over time, investigations into the development and legitimization of dedicated service units, and comparative studies across sectors, firm sizes, and digital service contexts.
- Finally, we strengthened the closing message of the paper. The revised conclusion emphasizes that successful CMS implementation depends less on the sophistication of condition monitoring technologies and more on the ability of OEMs to align customer value creation, service development practices, organizational capabilities, and change management processes. The concluding section now highlights how OEMs can transform condition monitoring technologies into sustainable service businesses through customer-centred value creation, legitimate service structures, multidisciplinary capabilities, and proactive management of organizational change.

We believe these revisions substantially improve the clarity, rigor, critical reflection, and practical relevance of the concluding section while providing a stronger synthesis of the study's contribution to CMS and digital servitization research.

**English Language and Clarity** We thank the reviewer for this observation and agree that the previous version of the manuscript would benefit from improvements in writing style, readability, and overall narrative flow. In response, we performed a comprehensive revision of the manuscript with a particular focus on language clarity and structure. The revisions included:

- Reducing the use of passive voice and rewriting numerous sentences using more direct and active constructions.
- Simplifying complex and lengthy sentences to improve readability and reduce ambiguity.
- Improving paragraph structure and logical flow, particularly in the Introduction, Theoretical Background, Findings, and Discussion sections.
- Replacing overly technical or jargon-heavy expressions with clearer terminology wherever possible while maintaining academic precision.
- Improving transitions between sections and paragraphs to strengthen the coherence of the overall argument.
- Reorganizing the Findings and Discussion sections to provide a clearer progression from challenges (RQ1), to solutions (RQ2), and finally to recommendations (RQ3).
- Strengthening the Discussion and Conclusions sections, ensuring that the narrative consistently links the empirical findings to the theoretical foundations and research questions.
- Revising the abstract, introduction, and conclusion to improve clarity, conciseness, and accessibility for a broader readership.

We believe that these revisions have substantially improved the clarity, consistency, and readability of the manuscript.

**Final Decision**

 We thank the reviewer for this comprehensive assessment and for identifying areas requiring substantial improvement. We carefully considered each of these concerns and have thoroughly revised the manuscript. The revised version differs substantially from the original submission in terms of theoretical framing, methodological transparency, presentation of findings, and discussion. Regarding theoretical foundation, we substantially strengthened the conceptual grounding of the study by repositioning CMS implementation within the digital servitization literature. The revised manuscript now explicitly adopts a socio-technical perspective (Münch et al., 2022) to interpret the implementation challenges experienced by OEMs. In addition, the literature review was expanded and updated to incorporate recent contributions on digital servitization. These revisions provide a stronger theoretical basis for understanding CMS implementation. Regarding methodological rigour, we substantially expanded the Methodology section. The revised manuscript now includes: a detailed justification of the expert elicitation approach; transparent expert selection criteria and participant profiles; a comprehensive description of the interview protocol; a detailed explanation of the thematic analysis procedure; a new section describing the progression from codes to first-order and second-order themes; a dedicated discussion of thematic saturation supported by a saturation table; an expanded discussion of analytical rigor, credibility, and trustworthiness. These additions significantly improve the transparency and reproducibility of the research process. Regarding the presentation and interpretation of results, the Findings section has been completely restructured. Rather than relying heavily on coding outputs, the revised manuscript organizes findings around the three research questions: implementation challenges (RQ1), solutions to overcome those challenges (RQ2), and recommendations for successful implementation (RQ3). The detailed coding structure was moved to the Extended Data (Data Analysis, Tables E1 to E4), while the manuscript now focuses on higher-level themes and analytical insights. In addition, the Discussion section was extensively rewritten to interpret the findings through the lenses of digital servitization literature. The Findings, Discussion, and Conclusions sections now explicitly address each research question individually and provide clear answers grounded in the empirical evidence. As a result, the revised manuscript now provides explicit and evidence-based answers to each research question, strengthening the coherence of the study and the support for its stated contributions. Regarding the claimed contributions, we clarified both the theoretical and managerial implications. The revised manuscript now explicitly explains that its contribution is not merely identifying implementation challenges, but providing empirically grounded, CMS-specific solutions and recommendations for OEMs. This distinction is important because much of the existing digital servitization literature offers generic recommendations, whereas our study translates these broader concepts into concrete implementation guidance for OEMs tailored to the particular characteristics of CMS. Finally, we conducted a comprehensive revision of the manuscript's language, structure, and readability.

 We are grateful for the reviewer's critical assessment, which contributed significantly to improving the quality, rigor, and contribution of the manuscript. We believe that the revised version addresses these concerns comprehensively and presents a substantially stronger study than the originally submitted manuscript. Collectively, these revisions strengthen the theoretical foundation, methodological rigor, structured argumentation, interpretive depth, and overall contribution of the manuscript.

# Reviewer report: Reviewer response for version 1

**DOI:** 10.21956/openreseurope.21680.r55266

**Published:** 2025-08-30

**Authors:** Erik Flores-García

**Affiliations:** 1 KTH Royal Institute of Technology, Stockholm, Sweden

**Brief summary of the manuscript**

 The manuscript presents a qualitative study focusing on the implementation of condition monitoring services (CMS) in the context of product service systems (PSS). It describes the challenges, potential solutions, and recommendations for successfully implementing CMS.

**Positive aspects**

 The manuscript provides relevant empirical insights that help synthesize previously identified theoretical concepts in PSS. By focusing on implementation, the authors offer a perspective of an early / frontloading effort with the potential of impactful outcomes to CMS. The grammar and structure of the manuscript is commendable. It communicates ideas effectively and arguments are presented in a logical flow.

 I thank the authors for their manuscript and the effort in conducting the study. The topic is highly relevant to smart manufacturing and the development of PSS. Below, I highlight my concerns for improving the quality of the study and point ways to overcome these concerns.

**Major concerns**

 A major concern includes the absence of a theoretical lens supporting the contributions of the study. This shortcoming affects the entire manuscript including its justification, the theoretical background, findings and contribution chapters.

 A theoretical lens is critical for contextualizing the research problem within existing knowledge and moving the field forward. Without a theoretical lens, interpreting findings may be surface-level, potentially missing deeper insights.

 Currently, the manuscript argues for the use of an inductive logic, which assumes that theory does not exist. I am not convinced by this assumption. Consider the section of Findings. Currently, this section works more like a review of literature positioned around the insight of interviewees. Literature about challenges, potential solutions and strategies for success do not seem to be in short supply based on the current content of this chapter.

 A way to overcome this short coming would be to modify the theoretical background. This section could provide deeper insight into the non-technical challenges of CMS / PSS. This aspect occupies only one paragraph but is the main focus of your study. Additionally, this section could also describe approach to potential solutions and successful implementation that may help interpret the findings of your interviews. Consider the following studies, their use of theory to interpret findings and position a theoretical contribution in qualitative studies:

 Münch, C., Marx, E., Benz, L., Hartmann, E., & Matzner, M. (2022). Ref 1

 Kohtamäki, M., Einola, S., & Rabetino, R. (2020). Ref 2

 The manuscript would greatly benefit from presenting these theoretical underpinning already in the theoretical background chapter. The findings section could then provide deeper insights about the novel findings of your qualitative data collection in comparison to existing literature.

 At the same time, the theoretical background could be better used to motivate / setup the research questions of the study. There is nothing wrong with the research questions but there is a need to justify why these are currently relevant in light of recent findings.

 The absence of a theoretical lens also has implications to the generalizability of your findings. The authors could benefit from describing the logic of their study that justifies generalizability of results and the use of theory to achieve it. Some ideas / examples of how to execute this in either an inductive or deductive logic in qualitative studies can be found in:

 Ketokivi, M., & Choi, T. (2014). Ref 3

 The concept of implementation is a cornerstone of the manuscript and one of the critical aspects for achieving a theoretical contribution. I do not think that the manuscript presents a sufficient conceptualization of implementation in the context of CMS or PSS. This problem could be addressed by offering an explicit definition grounded on the literature. The theoretical background should include a short synthesis of the constituent of implementation, its stakeholders, phases and implications to the end goals of PSS.

 Closely related to the concern above is the absence of a clear definition of the target group of the manuscript. At the beginning of the article, it appears that the main target group, those responsible for implementing CMS, include manufacturing companies developing PSS. The chapter including the Findings and afterwards present a change of this perspective. After this chapter it appears that the target group is both the companies developing PSS and the end users of a CMS. The manuscript would greatly improve by establishing clearly the target group and presenting a consistent narrative throughout the text.

 There is a need for improving the justification of an explorative study and its inductive reasoning.

 The distinction between PSS and CMS is unclear in the current version of the manuscript. Do the authors mean that PSS is a special case of CMS? Is CMS an initial step in PSS?

 The section of findings is very difficult to understand. One cannot distinguish between the voice of interviewees and literature. Additionally, this section presents a contradiction to the problem definition of the study. While problem definition states that there is limited literature on the topic, the findings section works more like a literature review of previous findings showing that there is in fact plenty of literature.

 The section on potential solutions presents a structure including value proposition, design, and requirements. Why not use this same structure from the beginning of the article to classify challenges, potential solutions, and recommendations? Why is this exclusively applied to the potential solutions? Is it not possible to provide theoretical underpinnings justifying the classification of solutions in relation to value proposition, design and requirements?

 The manuscript presents two contributions: a) an in-depth examination of implementation challenges and b) insights helping improve technological readiness and market success. A first problem with these is that they are presented for the first time very late in the text p. 16. Additionally, I am not convinced that manuscript achieves or intends to improve technological readiness. The argument followed throughout the manuscript is the focus on non-technological challenges of CMS.

**Minor concerns**

 Make sure to include the purpose of your study in the abstract. Additionally, the abstract would benefit from a clear description of its findings. This could include a clear description of the three major findings: challenges, potential solutions, and recommendations.

 Describe the background of the interviewees and their relevance from a PSS perspective.

 There is no in-text reference to the first and second order themes tables in the main section of the text for page 8 paragraph 2.

 Perhaps I missed something, but what is the difference between “our three main research questions” and the “main questions” described in the first paragraph of the section Findings: challenges in p. 8.

 The current version of the manuscript would improve by having separate paragraphs for each challenge described in findings section. Consider the current setup of the last paragraph of p. 8.

 The text would benefit from an extended analysis / description of the managerial contributions of the study. What stakeholders will benefit? What pitfalls will be avoided and how will this contribute to improving current industrial / business practices? How do the results compare or improve the current way of working of practitioners?

 The manuscript would benefit from a better description of its limitations leading to future steps.

Is the study design appropriate and does the work have academic merit?

Partly

Is the work clearly and accurately presented and does it cite the current literature?

Yes

If applicable, is the statistical analysis and its interpretation appropriate?

Not applicable

Are all the source data underlying the results available to ensure full reproducibility?

Yes

Are the conclusions drawn adequately supported by the results?

Partly

Are sufficient details of methods and analysis provided to allow replication by others?

Partly

Reviewer Expertise:

digital services in smart production logistics

I confirm that I have read this submission and believe that I have an appropriate level of expertise to confirm that it is of an acceptable scientific standard, however I have significant reservations, as outlined above.

## References

[1] : Capabilities of digital servitization: Evidence from the socio-technical systems theory. *Technological Forecasting and Social Change* .2022;176: 10.1016/j.techfore.2021.121361 10.1016/j.techfore.2021.121361

[2] : Exploring servitization through the paradox lens: Coping practices in servitization. *International Journal of Production Economics* .2020;226: 10.1016/j.ijpe.2020.107619 10.1016/j.ijpe.2020.107619

[3] : Renaissance of case research as a scientific method. *Journal of Operations Management* .2014;32(5) : 10.1016/j.jom.2014.03.004232-240 10.1016/j.jom.2014.03.004

# Response

**Published:** 2026-08-04

**Authors:** Ion Iriarte

**Affiliations:** Design Innovation Center, Mondragon University Higher Polytechnic School, Arrasate, Basque Country, Spain

We sincerely thank the reviewer for this insightful and constructive feedback. Following your recommendations, we have substantially revised the paper.

**Major concerns** **Introduction** We substantially revised the Introduction to provide a clearer and more coherent narrative linking Industry 4.0, digital servitization, and Condition Monitoring Services (CMS). We strengthened the justification for the study by explicitly discussing the disconnect between the predominantly technology-oriented CMS literature and the broader organizational, managerial, and business-related challenges associated with CMS implementation. In addition, we incorporated the digital servitization literature recommended by the reviewer, particularly the socio-technical perspective of Münch et al. (2022) and the paradox perspective of Kohtamäki et al. (2020), to establish a stronger theoretical foundation for the study. The revised introduction now contrasts the technology-focused CMS literature with the broader digital servitization literature, while also acknowledging that digital servitization research often examines digitally enabled services at a relatively general level and therefore provides limited guidance regarding the specific implementation challenges associated with CMS. This revised positioning provides a clearer research gap, stronger theoretical motivation, and a more explicit rationale for the study's research questions and contributions.

**Theoretical Background** We substantially revised and reorganized the entire theoretical background section.

 First, we restructured the literature review around thematic sections to improve readability and provide a clearer progression from the conceptualization of CMS to implementation challenges, implementation approaches, and the identification of the research gap. This new structure aligns the theoretical background more closely with the research questions and findings.

 Second, we clarified the concept of CMS implementation. The revised theoretical background now explicitly conceptualizes implementation as the organizational process through which OEMs develop, integrate, deliver, commercialize, and scale CMS-based service offerings within their digital servitization strategies.

 Third, we strengthened the theoretical foundations of the study by introducing a socio-technical systems perspective based on Münch et al. (2022). This perspective serves as the primary theoretical lens of the study and frames CMS implementation as the outcome of interactions between technological, organizational, and human capabilities rather than as a purely technological challenge. We also incorporated the servitization paradox perspective of Kohtamäki et al. (2020), enabling us to analyse CMS implementation as a process characterized by competing demands related to value creation, customer relationships, business model development, and organizational transformation. These revisions also enabled us to articulate a clearer theoretical contribution. Specifically, the revised manuscript demonstrates how CMS implementation challenges, solutions, and recommendations can be interpreted through a socio-technical digital servitization perspective, thereby extending current understanding of CMS implementation beyond its traditional technological orientation. Fourth, we expanded the discussion of CMS implementation challenges and moved beyond a descriptive review of prior studies. The revised manuscript now provides a more critical synthesis of the literature by examining challenges related to value proposition development, organizational capability building, customer collaboration, organizational transformation, and business model innovation. In particular, the service paradox is no longer mentioned only briefly but is analysed as a manifestation of broader tensions associated with value creation, service monetization, resource allocation, and organizational change.

 Fifth, we introduced a dedicated section reviewing approaches proposed in the literature for overcoming implementation challenges. This section synthesizes research on value creation, organizational capability development, paradox management, organizational transformation, business model innovation, leadership, and customer co-creation practices. This literature synthesis provides a stronger theoretical basis for interpreting the empirical findings and discussing potential implementation solutions.

 Finally, the research gap is now developed progressively throughout the theoretical background rather than being introduced only at the end. The revised manuscript acknowledges the valuable contributions of digital servitization research while arguing that existing studies often address digitally enabled services at a relatively high level of abstraction and provide limited guidance regarding the specific implementation conditions associated with CMS. This stronger positioning provides a clearer justification for the exploratory nature of the study and for the three research questions.

***Conceptualization of CMS implementation*** We agree that the concept of implementation was insufficiently defined in the original manuscript. Therefore, the revised manuscript explicitly conceptualizes CMS implementation as the organizational process through which OEMs develop, integrate, deliver, and scale CMS-based service offerings as part of their digital servitization strategy. This definition is positioned early in the theoretical background and used consistently throughout the manuscript.

***Clarification of CMS and PSS*** The relationship between CMS and PSS has also been clarified. In the revised manuscript, CMS are conceptualized as a specific type of Product-Service System in which value is co-created through the integration of physical products, IoT technologies, operational data, and service activities.

***Clarification of the target group*** We also addressed the reviewer's concern regarding the target group of the study. The revised manuscript consistently positions OEMs as providers of CMS offerings. While customer-related factors remain important because value co-creation is central to CMS implementation, the primary unit of analysis and intended audience of the study are now clearly defined as OEMs implementing CMS within their digital servitization strategies.

***Exploratory approach and generalizability*** Finally, we strengthened the justification of the exploratory design by incorporating the arguments of Ketokivi and Choi (2014) regarding qualitative inquiry and theory development in complex organizational settings. The revised manuscript now explains that the objective of the study is not statistical generalization but the development of analytically generalizable insights concerning CMS implementation challenges, solutions, and recommendations.

**Findings and Discussion** We thank the reviewer for the valuable observations regarding the readability and analytical structure of the Findings section. We agree that the previous version did not sufficiently distinguish empirical findings from literature-based interpretation. To address this concern, we substantially revised the manuscript by clearly separating the Findings and Discussion sections. The Findings section now focuses exclusively on the empirical evidence derived from the expert interviews, whereas engagement with prior studies, theoretical interpretation, and the articulation of contributions have been moved to the Discussion section. This revision makes it easier for readers to distinguish between the perspectives of the interviewees and the authors’ interpretation of the findings in relation to existing knowledge. Furthermore, the Discussion is now explicitly structured around RQ1–RQ3, providing clear answers to each research question and strengthening the link between the empirical results and the study objectives. We also clarified the research gap throughout the Introduction and Discussion. Our intention was never to argue that literature on CMS challenges and solutions does not exist, but rather that existing CMS research has predominantly focused on technological aspects of condition monitoring systems (e.g., diagnostics, prognostics, sensing technologies, and predictive analytics), whereas considerably less attention has been devoted to the organizational, and managerial challenges associated with implementing CMS. In addition, the revised manuscript now acknowledges that while digital servitization research provides valuable insights into organizational transformation and capability development, it often addresses digitally enabled services at a relatively high level of abstraction. Consequently, the Discussion now positions our findings both against the CMS literature and within the broader digital servitization literature to explain how the study addresses this gap and provides more context-specific insights for CMS implementation. The revised Discussion explicitly links these themes to digital servitization research, particularly the socio-technical perspective (Münch et al., 2022), service innovation and co-creation literature (Sjödin et al., 2020), value capture research in advanced services (Kamp et al., 2023), and the servitization paradox perspective (Kohtamäki et al., 2020).

 These theoretical connections help explain why the identified challenges emerge, how organizations can address them, and why they are particularly relevant within CMS implementation contexts. We thank the reviewer for highlighting the need to clarify the study’s contribution.

 We agree that the term technological readiness was not fully aligned with the actual focus and findings of the study.

 Accordingly, we revised the contribution statements throughout the manuscript, including the Introduction, Discussion, and Conclusions sections. The contributions are now introduced earlier in the paper and are consistently aligned with the study’s objectives and findings. More specifically, we removed references suggesting that the study contributes to improving technological readiness and instead emphasize that the study contributes to understanding the socio-technical dimensions of CMS implementation. The revised manuscript now explicitly argues that its contribution lies in identifying CMS-specific implementation challenges, proposing solutions to overcome them, and developing recommendations that translate broad digital servitization principles into more concrete implementation guidance for OEMs. We believe these revisions provide a more accurate representation of the study’s objectives, findings, theoretical contribution, and practical relevance.

**Minor concerns**

*Abstract* We have substantially revised the abstract to provide a clearer and more comprehensive overview of the study. Specifically, the revised abstract now explicitly states the purpose of the study, namely to investigate the challenges limiting CMS implementation, the solutions used to overcome these challenges, and the recommendations that support successful CMS implementation by OEMs.

*Background of the interviewees and their relevance from a PSS perspective* We agree that the original manuscript did not sufficiently explain the relevance of the selected experts. To address this issue, we introduce a more detailed description of the expert selection criteria and the relevance of each participant to the phenomenon under investigation. In particular, we expanded Table 1 to include the professional background and specific relevance of each expert to CMS implementation.

*In-text references* We thank the reviewer for identifying this omission. In the revised manuscript, we have improved the consistency between the narrative findings and the coding structure. Specifically, explicit references to the first-order and second-order theme tables have been incorporated throughout the Findings section. *The “Three main research questions” vs the “main questions”* We thank the reviewer for highlighting this source of confusion. We agree that the terminology used in the previous version could suggest the existence of two different sets of questions. In reality, the “main questions” referred to in the Findings section corresponded directly to the three research questions presented earlier in the manuscript. To improve clarity, we have revised the wording throughout the manuscript and now consistently refer only to the three research questions (RQ1–RQ3).

*Separate paragraphs for each challenge* We thank the reviewer for this helpful suggestion. To address this issue, we substantially restructured the Findings section. The revised manuscript now presents the results through separate subsections and paragraphs corresponding to each major challenge category identified in the analysis. Specifically, challenges are now discussed separately under the themes of CMS value proposition, CMS design process, and organizational and customer readiness, with each challenge receiving its own dedicated explanation.

*Managerial contributions and limitations leading to future steps* We thank the reviewer for these valuable suggestions. In response, we substantially revised the Conclusions, Limitations and Future Research section to strengthen both the managerial implications and the discussion of limitations. Regarding the managerial contributions, we further clarified how the findings can improve current industrial practice by helping OEMs avoid common implementation pitfalls such as: technology-centred value propositions, insufficient customer involvement, fragmented ownership of CMS initiatives, weak service-oriented capabilities, and limited value quantification and value capture mechanisms. We also expanded the managerial implications to explain how the proposed recommendations differ from and improve current practices observed by the experts. Regarding the limitations, we expanded the discussion beyond a brief acknowledgment of methodological constraints. The revised manuscript now critically reflects on the implications of relying on expert perceptions, the focus on OEMs and CMS contexts, the absence of multiple data sources and formal participant validation procedures, and the cross-sectional nature of the study. Furthermore, we explicitly connect these limitations to future research opportunities by proposing longitudinal studies, multi-stakeholder investigations, and research exploring the development of organizational legitimacy and dedicated service units in manufacturing organizations.

 This revision now provides a clearer link between the study's limitations and future research directions.

 We are grateful for the reviewer’s thoughtful and constructive comments, which have significantly strengthened the theoretical grounding, conceptual clarity, methodological justification, and overall contribution of the manuscript. We believe the revised version now provides a more coherent, theoretically informed, and practically relevant account of CMS implementation within the context of digital servitization.
